# Dissection of quantitative trait nucleotides and candidate genes associated with agronomic and yield-related traits under drought stress in rapeseed varieties: integration of genome-wide association study and transcriptomic analysis

**DOI:** 10.3389/fpls.2024.1342359

**Published:** 2024-03-19

**Authors:** Maryam Salami, Bahram Heidari, Bahram Alizadeh, Jacqueline Batley, Jin Wang, Xiao-Li Tan, Ali Dadkhodaie, Christopher Richards

**Affiliations:** ^1^ Department of Plant Production and Genetics, School of Agriculture, Shiraz University, Shiraz, Iran; ^2^ Oil Crops Research Department, Seed and Plant Improvement Institute, Agricultural Research Education and Extension, Organization, (AREEO), Karaj, Iran; ^3^ School of Biological Sciences, University of Western Australia, Perth, WA, Australia; ^4^ School of Life Sciences, Jiangsu University, Zhenjiang, China; ^5^ United States Department of Agriculture (USDA), Agricultural Research Service (ARS), National Laboratory for Genetic Resources Preservation, Fort Collins, CO, United States

**Keywords:** drought, gene ontology, linkage disequilibrium, QTN, RNA-sequencing, single-nucleotide polymorphism, yield

## Abstract

**Introduction:**

An important strategy to combat yield loss challenge is the development of varieties with increased tolerance to drought to maintain production. Improvement of crop yield under drought stress is critical to global food security.

**Methods:**

In this study, we performed multiomics analysis in a collection of 119 diverse rapeseed (*Brassica napus* L.) varieties to dissect the genetic control of agronomic traits in two watering regimes [well-watered (WW) and drought stress (DS)] for 3 years. In the DS treatment, irrigation continued till the 50% pod development stage, whereas in the WW condition, it was performed throughout the whole growing season.

**Results:**

The results of the genome-wide association study (GWAS) using 52,157 single-nucleotide polymorphisms (SNPs) revealed 1,281 SNPs associated with traits. Six stable SNPs showed sequence variation for flowering time between the two irrigation conditions across years. Three novel SNPs on chromosome C04 for plant weight were located within drought tolerance-related gene *ABCG16*, and their pleiotropically effects on seed weight per plant and seed yield were characterized. We identified the C02 peak as a novel signal for flowering time, harboring 52.77% of the associated SNPs. The 288-kbps LD decay distance analysis revealed 2,232 candidate genes (CGs) associated with traits. The CGs *BIG1*-*D*, *CAND1*, *DRG3*, *PUP10*, and *PUP21* were involved in phytohormone signaling and pollen development with significant effects on seed number, seed weight, and grain yield in drought conditions. By integrating GWAS and RNA-seq, 215 promising CGs were associated with developmental process, reproductive processes, cell wall organization, and response to stress. GWAS and differentially expressed genes (DEGs) of leaf and seed in the yield contrasting accessions identified *BIG1-D*, *CAND1*, and *DRG3* genes for yield variation.

**Discussion:**

The results of our study provide insights into the genetic control of drought tolerance and the improvement of marker-assisted selection (MAS) for breeding high-yield and drought-tolerant varieties.

## 1 Introduction

Rapeseed (*Brassica napus* L.) is a member of the *Brassicaceae* family and is ranked second in global oilseed production (Meyer and Purugganan, 2013; FAO, 2018; Raboanatahiry et al., 2018). It is utilized almost entirely for oil production, food, feedstock, and biodiesel production. Rapeseed, originated from interspecific hybridization between turnip rape (*B*. *rapa*, AA, 2n = 20) and cabbage (*B*. *oleracea*, CC, 2n = 18), is the most widespread oilseed crop in various climates due to the ability to germinate and grow at low temperatures (Ren et al., 2000; Chalhoub et al., 2014; Koh et al., 2017; Wozniak et al., 2019).

Understanding the genetic bases of yield-related trait is of great significance for breeding high-yield rapeseed (Shi et al., 2011; Khan et al., 2018; Raboanatahiry et al., 2018; Zhu et al., 2021). Although both spring and winter growth habit forms have been identified in rapeseed, the winter form has shown a higher grain yield (Fordonski et al., 2016). The grain yield of rapeseed can be directly increased through selection for fecundity and by indirect selection for phenological traits which show complicated genetic control in plants (Nowosad et al., 2016; Marjanovic-Jeromela et al., 2019). Nevertheless, the polygenic nature of the genetic control of yield and its components and the influence of environmental variables complicates mining genetic loci contributing to stress tolerance in plants including rapeseed. Water-deficit stress in the period between the flowering stage and pod formation stages causes up to around 30% loss in grain yield (Elferjani and Soolanayakanahally, 2018).

Drought stress in arid and semiarid areas restricts plant growth and production in agriculture (Haq et al., 2014; Seleiman et al., 2021). At the early vegetative growth stage, drought slows down the rapeseed growth by inhibiting cell expansion and division (Yosefi and Heidari, 2022). However, drought at the reproductive development stage could drastically reduce rapeseed yield by producing small and medium-sized grains (Hatzig et al., 2018). Several studies have been conducted to evaluate the drought tolerance of rapeseed (Yarnia et al., 2011; Liu et al., 2015; Zhou et al., 2021). However, progress in improvement for drought tolerance is slow because of the complex genetic architecture of drought stress tolerance controlled by several minor and major genes (Bernardo, 2008). Understanding the genetic control of drought tolerance mechanisms can significantly accelerate the development of drought-tolerant varieties through marker-assisted selection (MAS) and genomic selection (GS).

Genome-wide association studies (GWASs) are currently the powerful tool to detect marker–trait associations (MTAs) and can be applied to mapping and identifying linked markers and candidate genes contributing to drought tolerance (Xiao et al., 2017). In rapeseed, GWAS has been used to identify loci and candidate genes for drought stress tolerance (Zhang et al., 2015; Khanzada et al., 2020; Shahzad et al., 2021; Salami et al., 2024). In a study consisting of 157 genotyped inbred rapeseed cultivars, GWAS was used to identify 320 SNPs linked with both seed weight (SW) and silique length (SL) traits and mapped to the gene *BnaA.ARF18* (Dong et al., 2018). In another study, 197 candidate genes were detected for budding, bolting, day to flowering (DTF), and the interval between DTF and bolting in rapeseed of which *FRIGIDIA* (*FRI*), *FLOWERING LOCUS C* (*FLC*), and *AGAMOUS-like 16* (*AGL16*) showed significant contribution to flowering time (Helal et al., 2021). Raman et al. (2019) conducted an association analysis in canola accessions using 11,804 SNPs under normal irrigation and water-stressed conditions and identified 47 SNPs on chromosome A02, and an additional 13 SNPs on chromosome C03 were associated with flowering close to *FLOWERING LOCUS T* (*FT)* and *FLOWERING LOCUS C* (*FLC*) genes for the drought avoidance mechanism (Raman et al., 2020). In the Hu et al. (2022) study, 628 SNPs were identified for 56 agronomically important traits through GWAS in a panel of diverse rapeseed accessions. A whole-genome resequencing and multilocus genome-wide association study (ML-GWAS) in rapeseed accessions revealed that 908 SNPs for agronomic and phenological traits of which 79 candidate genes were associated with *BnaA09g39790D* (*RNA helicase*), *BnaA09g39950D* (*Lipase*), and *BnaC09g25980D* (*SWEET7*) genes (Zhang et al., 2023a).

Transcriptomics and RNA-seq technology exploits transcript sequences to estimate patterns of gene expression, alternative splicing, and allele-specific expression (Marguerat and Bahler, 2010; Zhang et al., 2023b). Transcript analysis can complement QTLs identified. In one study, RNA-seq was performed on eight tissues of extremely high- and low-harvest index (HI) rapeseed accessions and demonstrated that 33 functional candidate genes were located within the confidence intervals of significant SNPs associated with HI-related traits (Lu et al., 2016). In another study, Zhang et al. (2023a) performed GWAS and transcriptome analysis for seed yield and yield-related traits in *Brassica napus* for identification of differentially expressed genes (DEGs) in the seed of contrasting seed size/seed weight accessions.

Expanding rapeseed production areas through cultivation of drought-tolerant varieties could be an efficient strategy to alleviate the adverse effects of drought. Genomic studies focused on drought tolerance can be translated into breeding objectives for varietal development. In the present study, we aimed to identify novel SNPs and key genes for drought tolerance related traits in rapeseed. First, we investigated the effects of drought stress on agronomic, grain yield-related, and yield-related traits. Then, a GWAS approach was used to determine novel SNPs/genes for traits in the well-watered and drought stress conditions in 3 years. The RNA-Sequencing (RNA-Seq) experiment was performed in the leaf and seed of the contrasting high- and low-yielding varieties to validate the genes associated with the position of the identified linked SNPs. Differentially expressed genes (DEGs) in the leaf and seed of the accessions in the yield contrasting varieties to explore the genetic basis of drought tolerance can be facilitated by integrated functional genomic approaches.

## 2 Materials and methods

### 2.1 Plant materials, field experiment, and drought treatments

The plant materials used in this study consisted of 119 rapeseed varieties including breeding lines, hybrids, and commercial cultivars provided by the Institute of Seed and Plant Improvement (SPII), Iran (**Additional File 1**). The field experiment was performed at the Research Farm of Plant Production and Genetics, Shiraz, Iran in a 3-year trial in 2017, 2018, and 2020 growing seasons. Plants growth was incomplete in a 2019 trial which was due to spring frost damage. The texture of the soil was silty loam, and concentrations of micro- and macronutrients are shown in **Additional File 2**. Seasonal temperature, relative humidity, and mean precipitation are shown in **Supplementary Figure S1**. The experimental design was a lattice by patterning 11 × 11-unit cells (11 varieties and 11 units per block) with three replicates per watering regime [well-watered (WW) and drought stress (DS)]. The plot size was 1 m^2^ with the between-plot distances of 0.5 m. Each plot was composed of four 1-m-long rows, each with 25 plants per 4-cm spaces. The seeds were sown on four rows 1 m in length on 17 September for the three seasons.

The fertilizers were applied at the rates of 250 kg N ha^−1^, 100 kg P ha^−1^, and 100 kg K ha^−1^. Phosphorus and potassium fertilizers were incorporated to the soil prior to sowing, and nitrogen was used as top dressed in different growth stages of rapeseed. In the well-watered condition, irrigation was carried out from planting till seed physiological maturity (maximum seed dry weight) as previously described by Ozer (2003). In the drought stress treatment, irrigation continued till the 50% pod development stage when irrigation then stopped till end of the growing season, which made three irrigation practices less in the drought-stressed plants than in the well-watered plants throughout the growing season. The numbers of irrigations in WW and DS treatments were 9 and 6, respectively.

For weed control, 3 L ha^−1^ Treflan^®^ HFP herbicide was sprayed at sowing and hand weeding was also followed during the growing season. The Pirimor 50 pesticide at a rate of 2 L ha^−1^ was used for the aphids on rapeseed at the flowering and early podding periods. Harvesting time was the first week of July when the siliquae in terminal raceme turned creamy white in color.

### 2.2 Measurement of phenotypic characteristics

Days to flowering (DTF) was measured as the interval between the time of sowing and the time when the first flowers opened on 50% of the plants followed by Matar et al. (2021) description. Days to silique development (DTSD) was recorded as a number of days from the sowing to the time that first pods appeared on 50% of the plants. Days to ripening (DTR) was measured as the interval between the dates of sowing and the time when pods were dried. Plant height (PH) (cm) was recorded from the ground to the tip of the main pod at the ripening stage. For branch number/plant (BNPP) and yield components including silique length (SL; cm), seed number/silique (SNPS), seed weight/plant (SWPP; g), and thousand seed weight (THSW; g), 10 randomly plants were harvested from the middle rows to avoid border effects in each plot. At the harvesting time, plants in two middle rows were cut for plant weight (PW; g), seed yield (SY; kg ha^−1^), and harvest index (HI; %) measurements. The grain weight of 10 spikes was used as grain weight per spike. The HI was calculated by dividing the grain yield by the biological yield.

### 2.3 Analysis of variance and estimation of genetic variation and genetic gain

Descriptive parameters including mean and standard deviation (SD) were calculated for each treatment in SAS software (version 9.4). Box and whisker plots were used for the graphical presentation of the descriptive statistics. The packages ggplot2 in R (version 4.3.2) for win (http://CRAN.R-project.org/, accessed on 23 February 2021) and RStudio (version 1.3.1093) (https://rstudio.com/, accessed on 23 February 2021) were used for analysis of boxplots (McGill et al., 1978; Wickham, 2016). The correlation matrix between variables and a constructing heat map of correlation coefficients were computed using packages plotly, heatmaply, and ggcorrplot (https://cran.rproject.org/web/packages/ggcorrplot/index.html) in the R software.

The PROC GLM procedure was used for combined analysis of variances (ANOVA) in the Statistical Analysis System (SAS) software (SAS Institute Inc., Cary, NC, USA, version 9.3) (Dodig et al., 2008). The RANDOM statement with the TEST as option procedure was used to define the year as a random effect and water treatment and genotype as fix effects in ANOVA.

Phenotypic and genotypic variances were calculated as shown in Equation 1 using the expected mean squares (EMS) of sources of variations in ANOVA as follows (Lush, 1949),


(1) $$\sigma_{ɡ}^{2}=(\frac{(\text{MSG}-\text{MSE})}{r})\times \left(100\right)$$

where $\sigma {ɡ}^{2}\;$ is the genotypic variance, MSG is the mean square for genotype, MSE is the error mean square in ANOVA, and r is the number of replications.


(2) $$\sigma_{p}^{2}=\sigma_{ɡ}^{2}+\sigma_{e}^{2}$$

where $\sigma_{p}^{2}$ and $\sigma_{e}^{2}\;$ are the phenotypic and environmental variances, respectively (Equation 2).

The environmental, genotypic, and phenotypic coefficients of variation were calculated as shown in Equations 3, 4, and 5 using as follows (Burton, 1952):


(3) $$\text{ECV}=\frac{MSE}{\bar{\text{x}}}\times (100)$$

(4) $$\text{GCV}=\frac{(\sigma_{ɡ})}{\bar{\text{x}}}\times \left(100\right)$$

(5) $$\text{PCV}=\frac{(\sigma_{p})}{\bar{\text{x}}}\times \left(100\right)$$

where ECV is the environmental coefficient of variation, GCV is the genotypic coefficient of variation, PCV is the phenotypic coefficient of variation, $\sigma_{ɡ}$ is the root of genotypic variance, $\sigma_{p}$ is the root of phenotypic variance, and x̅ is the trait mean.

Variance components were used to calculate the broad-sense heritability ( $h^{2}$ ) in Equation 6 as follows (Marwede et al., 2004):


(6) $$h^{2}\;=\frac{\sigma_{ɡ}^{2}}{\sigma_{p}^{2}}\;\times \left(100\right)$$

Simple genetic advance (GA) and GA over means (GAM) were calculated as shown in Equations 7 and 8 as follows::


(7) $$\text{GA}=(k\times h^{2}\times \sigma_{p}/\bar{x})$$

(8) $$\text{GAM}=(GA/\bar{x})\;\times \left(100\right)$$

where *k* is the selection intensity which was 1.76 denoting selection of 10% of top-ranked varieties, $h^{2}$ is heritability in a broad sense, and x̅ is the trait mean.

### 2.4 Reference mapping and variant calling

Pseudo-genome sequences of the diploid A (283.8 Mb) and C genomes (488.6 Mb) were combined and used as the reference sequences for mapping analyses. Reads for each genotype were aligned independently to the reference genome using CLC Genomics Workbench (version 7.0.4). The mapped reads were interrogated for sequence variation using the CLC Bio probabilistic variant calling tool. A minimum depth of coverage of 3× for 454 and 8× for Illumina data was required for SNP calling. Mapping data and variant calls were exported from CLC and combined using a custom Perl script to determine reference, or variant call for every genotype at all variant positions.

### 2.5 DNA extraction and single SNP-based association mapping

Genomic DNA was isolated from the fresh leaves collected from a bulk of five randomly chosen plants per variety in a greenhouse (Murray and Thompson, 1980). The genomic DNA was quantified using a NanoDrop ND-1000 Spectrophotometer (NanoDrop Technologies, Inc., Wilmington, DE, United States). The DNA samples were used for genotyping *Brassica* 60 K Infinium array as described in the manufacturer’s protocol (Illumina Inc., San Diego, CA). Quality preprocessing of 52,157 SNPs obtained from 60K chips was done by using TASSEL software v5 (Bradbury et al., 2007). The SNPs were filtered for site coverage (90%), minimum minor allele frequency (MAF) of 0.05 with only biallelic markers, and low rates of missing data (≤10%) using the TASSEL (version 5).

### 2.6 Population genetics analysis, linkage disequilibrium, and LD decay

The polymorphism information content (PIC) value of each SNP locus in all varieties and the PIC values on each chromosome were calculated by PowerMarker (version 3.2.5) (Liu and Muse, 2005). We generated 29,310 SNPs involving 119 varieties derived from Iran, Germany, France, America, Australia, Hungary, Serbia, and Russia (**Additional File 1**). For population structure analysis, the natural logarithms of probability data (LnP(K)) and the *ad hoc* statistic ΔK were calculated (Su et al., 2017). The population structure underlying the collection of rapeseed varieties was analyzed using STRUCTURE (version 2.3.4) (Hubisz et al., 2009). The model-based Bayesian cluster analysis program was used to identify subpopulations. A total 10,000 burn-in periods followed by 100,000 Markov Chain Monte Carlo (MCMC) iterations from K = 3 to K = 10 were used to identify the optimal number of clusters (K). Three independent runs were generated for each K. The results were collated by the Structure Harvester tool (Earl and VonHoldt, 2011), and the best K-value was identified based on the delta K method (Evanno et al., 2005). A neighbor-joining (NJ) tree was created to validate population stratification with the software TASSEL (version 5). A PCA was done on the significant SNPs data using R software (version 4.3.2) (R Core Team, 2018) with the ggplot2 (Wickham, 2016) and ape (Paradis and Schliep, 2018) packages, respectively. To investigate chromosome-wide and the genome-specific patterns of linkage disequilibrium (LD) (*r^2^*), the software TASSEL (version 5) (www.maizegenetics.net/) with 1000 permutations was used. After quality control processing, a total of 29,310 high-quality SNPs with MAF ≥0.05 and pairwise *r^2^* values were used to determine the extent of LD decay across genome and among chromosomes.

### 2.7 Genome-wide association analysis

The marker–trait associations (MTAs) were analyzed using the program TASSEL (version 5). Four models, namely, general linear model (GLM) with the Q matrix of population structure (GLM + Q), mixed linear model (MLM) with both the kinship (K) as a random effect and Q matrices (MLM + Q), GLM model with the major principal components (PC) matrix (GLM + PC) and MLM with both the PC and K matrices (MLM + K + PC), were used to identify the MTAs. In GWAS, five PCs based on their cumulative eigenvalue contribution were used in population structure analysis. The Q matrix obtained from structure analysis and the relative kinship and PC matrices were calculated by TASSEL software. The phenotypic variation explained by significant SNP marker (*R^2^*) was calculated in TASSEL (version 5) (Bradbury et al., 2007). Quantile–quantile (QQ) plots were shown with −log10(*P*) of each SNP and expected *P*-value using the R package qqman (https://cran.rproject.org/web/packages/qqman/index.html). Manhattan plots were drawn in TASSEL software.

### 2.8 Screening candidate genes overlapped with the SNP position

To identify candidate genes (CGs) related to the SNPs of traits under the well-watered and drought-stressed conditions, the flanking sequences of the linked SNPs obtained from the “Darmor-bzh” reference genome (http://www.genoscope.cns.fr/brassicanapus/data/) was used to search in the rapeseed genome by Ensembl Plants (https://plants.ensembl.org/). Consequently, all the genes underlying the genomic region of each SNPs were functionally annotated by Ensembl Plants (https://plants.ensembl.org/) and online resources (https://genome.ucsc.edu/ and https://www.ncbi.nlm.nih.gov/). The CGs were identified based on their putative function in rapeseed or closely related species.

### 2.9 Allele effect and haplotype analyses

The allele effects for the linked significant SNPs were analyzed as previously described by Alemu et al. (2021). Varieties were divided into two different groups according to their specific SNP alleles, and the means were compared using Turkey’s honest significant difference (HSD) test. Exploring and harnessing haplotype diversity helps in the detection of CGs for improvement of target traits in crops (Qian et al., 2017). A haplotype association test was performed to investigate the combined effect of the linked significant SNPs. The SNPs in the same haploblock and the LD of significant SNPs were determined using Haploview (version 4.2) (Barrett et al., 2005). A standardized disequilibrium coefficient (D′) was used to evaluate the LD between markers and generate the LD heatmap. Haploid blocks were detected based on LD using the confidence intervals (CI) method in Haploview (version 4.2) (Gabriel et al., 2002).

### 2.10 RNA extraction, library construction, and RNA-sequencing

To validate the GWAS-identified SNPs contributed to drought tolerance, the expression of candidate genes associated with the position of the related SNPs was analyzed in two drought-tolerant and two drought-sensitive varieties. The experiment consisted of the RNA-Seq analysis in two top high- (G19 and G41) and two top low- (G111 and G114) yielding varieties showing contrasting yield under the WW and DS conditions. The plants were grown in 20-cm-diameter pots in the greenhouse under 12-h light/12-h dark conditions with normal experimental management. There were six plants in each pot. When the flower buds became visible, plants were randomly divided into two groups, each with three plants: the control group and the drought stress treatment group and each experiment underwent three biological replicates. During irrigation, drought-treated flower pots maintain a soil moisture content of 10% (irrigated with PEG6000 at a concentration of 20%), whereas well-watered flower pots maintain a soil moisture content of 30% (irrigated with sterile water of equal volume). Thirty days after flowering, the leaf and mature seeds were harvested from plants in each replicate group and each condition (the well-watered and drought stress treatments). All harvested seeds and leaves were immediately frozen using liquid nitrogen and transferred to a deep freezer (−80°C) for storage.

A total of 24 samples (control and treatments with three biological replicates, respectively) were prepared for RNA-Seq. Total RNA was extracted from the seeds and leaves using a Plant RNA Mini Kit (Tiangen, Inc., China) according to the manufacturer’s instruction. Four cDNA libraries were constructed, and RNA-Seq was performed on a DNBSEQ-G400 platform.

Low-quality reads were filtered out using the NGS QC toolkit (version 2.2.3) (https://omictools.com/ngs-qc-toolkit-tool) (Patel and Jain, 2012). High-quality reads from the raw sequencing reads were matched to the *B*. *napus* reference genome of “Darmor-bzh” (http://www.genoscope.cns.fr/brassicanapus/). The identified genes in the previous step were quantitatively analyzed using Cluffquant and Cluffnorm of Cufflinks 2.0.0 (http://cole-trapnell-lab.github.io/cufflinks/releases/v2.0.0/).

### 2.11 Identification of differentially expressed genes

The DEGs were identified based on FPKM (fragments per kilo base of transcript per million mapped fragment) and Q value (<0.05) (Q value: error-corrected value after multiple testing), and a log_2_ (fold change) ≥1 was set as the threshold to identify the significance of gene expression differences. Furthermore, to verify the statistical significance and hierarchical clustering of DEGs, a heat map was generated using R software (version 4.3.2).

### 2.12 Enrichment analyses of Gene Ontology and Kyoto Encyclopedia of Genes and Genomes pathways

To further understand the function of DEGs, we performed Kyoto Encyclopedia of Genes and Genomes (KEGG) and Gene Ontology (GO) analyses on the identified DEGs. The sequence file of each gene was used as input into Eggnog software (version 2.0.1) to identify annotation of genes (Huerta-Cepas et al., 2017). GO and KEGG analyses were conducted using the ClusterProfiler (version 4.0.0) R package. Only GO terms or KEGG pathways with *P-*value <0.05 verified for subsequent analyses. The REVIGO program (http://revigo.irb.hr/) was used to remove redundant GO terms (Supek et al., 2011).

### 2.13 Integration of genome-wide association study and transcriptome data

The RNA‐seq data were used for the ratios of genome‐wide up‐ and downregulated DEGs. In addition, we calculated the ratios of up‐ and downregulated DEGs within the 288-kbp intervals corresponding to the significant SNPs of the GWAS analysis. Then, we compared the DEG ratios for all genome‐wide genes and for potential drought tolerance-related genes detected in the GWAS of the high- and low-yield contrasting varieties under drought stress.

## 3 Results

### 3.1 Phenotypic variation and heritability of traits

**Figure 1:**
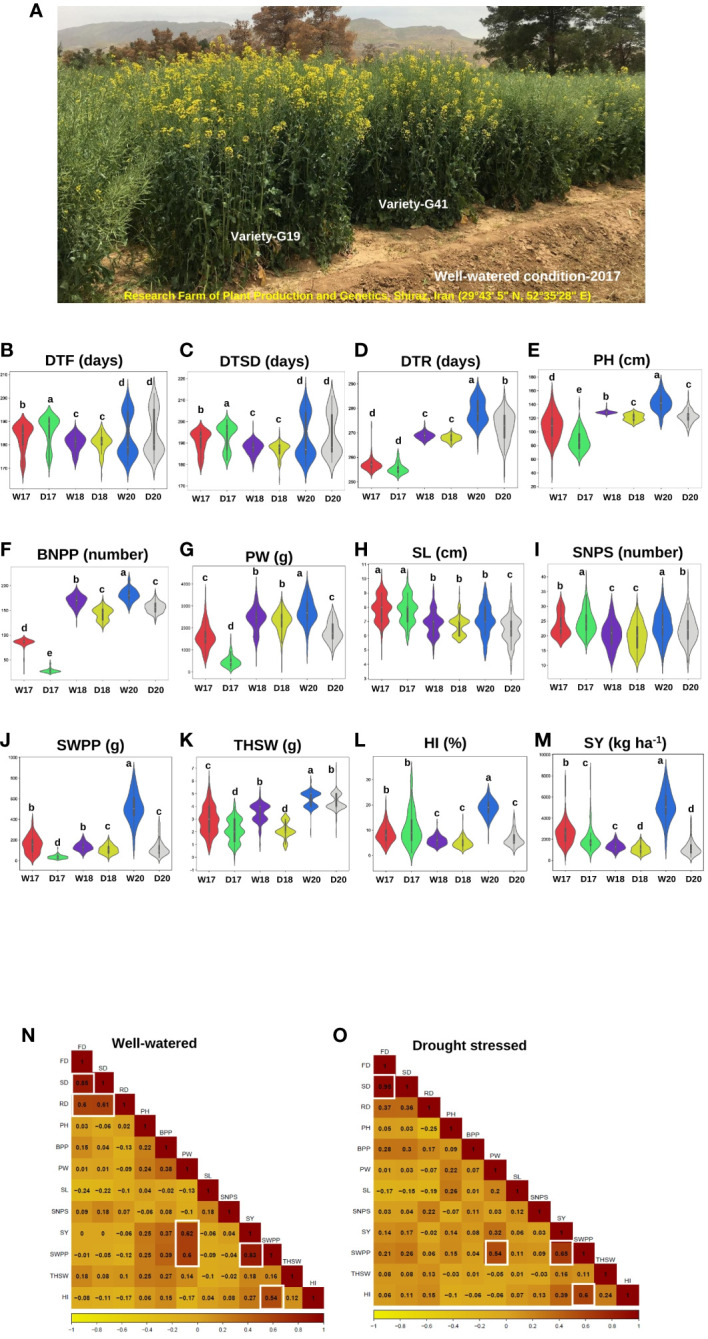
Agronomic and yield-related traits phenotyping in rapeseed (*Brassica napus* L.) varieties under two watering regimes (well-watered and drought stress conditions) across three years, 2017, 2018, and 2020. **(A)** A diverse collection of rapeseed varieties was assessed for agronomic and yield-related traits under well-watered conditions in 2017 in Research Farm of Plant Production and Genetics, Shiraz, Iran (29°43′ 5″ N, 52° 35′28″ E), highlighting the range of phenotypic diversity within the panel. G19 and G41 were high-yield varieties. **(B-M)** The violin plots illustrating the changes in agronomic and yield-related traits under well-watered and drought stress conditions from the individual data sets of 3 years (2017, 2018, and 2020) of 119 rapeseed varieties. The width of the violin plot represents the density of the distribution. The white dot in the box plot shows the median value, and the upper and lower boxes in the box represent the upper and lower quartiles of the data set. Data are means ± SD, *P*-value < 0.05, as determined by multiple comparison testing by one-way ANOVA. Traits represent as **(B)** days to flowering (DTF), **(C)** days to silique development (DTSD), **(D)** days to ripening (DTR), **(E)** plant height (PH), and **(F)** branch number/plant (BNPP), **(G)** plant weight (PW), **(H)** silique length (SL), **(I)** seed number/silique (SNPS), **(J)** seed weight/plant (SWPP), **(K)** thousand seed weight (THSW), **(L)** harvest index (HI), and **(M)** seed yield (SY). WW17, DS17, WW18, DS18, WW20, and DS20 were the codes of the two watering regimes during 3 years: well-watered in 2017, drought stress in 2017, well-watered in 2018, drought stress in 2018, well-watered in 2020, and drought stress in 2020, respectively. **(N, O)** Heat map showing the correlation between the agronomic and yield-related traits under two watering regimes in three growing seasons (2017, 2018, and 2020). **(N)** Well-watered condition, **(O)** drought stress condition. Traits represent as flowering date (FD), silique date (SD), ripening date (RD), plant height (PH), branches per plant (BPP), plant weight (PW), silique length (SL), seed number/silique (SNPS), seed yield (SY), seed weight/plant (SWPP), thousand seed weight (THSW), and harvest index (HI). A color scale showing the correlation values ranging from dark yellow, −1, to orange, 0, to 1, dark red is shown below the heat map.

The results of ANOVA for the main effects of treatments and the interactions are shown in **Additional File 3**. The effects of year (Y), environment (E), and genotype (G) were statistically significant (*P*-value < 0.01), which shows variation among varieties for traits over years and irrigation treatments. The G × Y, G × E, Y × E, and Y × G × E interactions were significant for all traits. Phenotypic variation for traits under normal irrigation and drought stress conditions is shown in **Table 1**; **Figures 1A–M**. The results showed that PW, SWPP, HI, and SY had high variation among the 119 rapeseed varieties in two irrigation regimes.

**Table 1:** Phenotypic variation of agronomic and yield related traits in 119 rapeseed (*Brassica napus* L.) varieties.

Trait	Watering regime	Mean	SD	Min	Max	ECV (%)	PCV (%)	GCV (%)	*h^2^* (%)	GA	GAM (%)
**DTF**	WW17	183.01	5.82	168	189	2.66	3.18	4.15	58.73	7.84	4.29
	DS17	186.00	6.34	171	192	1.20	3.39	3.59	88.91	10.45	5.62
	WW18	180.64	3.57	171	188	0.89	1.98	2.17	83.26	5.74	3.18
	DS18	180.62	4.13	166	187	0.48	2.23	2.28	95.65	6.93	3.83
	WW20	183.54	9.03	171	201	0.54	4.95	4.98	98.82	15.91	8.67
	DS20	184.89	9.14	172	202	0.83	4.98	5.05	97.31	16.00	8.65
**DTSD**	WW17	190.73	4.97	179	197	2.02	2.56	3.26	61.70	6.75	3.54
	DS17	193.04	5.81	182	190	1.15	3.01	3.22	87.19	9.54	4.94
	WW18	187.73	3.18	179	198	0.56	1.67	1.76	89.81	5.22	2.78
	DS18	186.88	3.45	173	195	0.55	1.79	1.87	91.39	5.62	3.01
	WW20	192.47	10.02	179	213	0.92	5.22	5.30	96.97	17.40	9.04
	DS20	193.72	9.71	181	212	0.95	5.01	5.10	96.52	16.77	8.66
**DTR**	WW17	256.84	2.27	253	273	0.79	0.87	1.17	54.99	2.91	1.13
	DS17	255.31	2.15	252	262	0.56	0.84	1.01	69.02	3.12	1.22
	WW18	268.80	1.46	266	274	0.42	0.54	0.68	62.46	2.01	0.75
	DS18	267.80	1.28	265	271	0.39	0.48	0.61	60.14	1.74	0.65
	WW20	278.58	4.84	265	289	0.33	1.75	1.78	96.64	8.41	3.02
	DS20	272.82	5.60	254	285	0.28	2.03	2.05	98.14	9.64	3.53
**PH**	WW17	108.20	19.35	41.33	157.55	6.11	18.04	19.05	89.73	32.55	30.08
	DS17	86.58	14.25	61.14	141.33	12.16	16.56	20.54	64.97	20.33	23.49
	WW18	178.24	2.37	123.45	141.33	0.84	0.78	1.15	46.38	2.80	0.94
	DS18	170.60	5.09	111.11	130.45	1.06	1.77	2.06	73.47	7.74	2.67
	WW20	142.31	12.50	113.34	173.12	2.09	8.81	9.05	94.69	21.47	15.09
	DS20	122.21	8.80	100.43	154.32	2.15	7.18	7.50	91.80	14.81	12.11
**BNPP**	WW17	84.21	9.92	29	92	7.09	11.76	13.73	73.31	14.92	17.72
	DS17	28.10	4.16	23	47	8.19	14.69	16.82	76.28	6.35	22.58
	WW18	168.53	12.20	125	193	4.70	7.37	8.75	71.06	18.43	10.94
	DS18	143.06	12.13	115	172	4.75	8.55	9.78	76.43	18.83	13.16
	WW20	181.23	12.35	149	218	1.85	6.94	7.18	93.34	21.39	11.80
	DS20	156.10	10.48	129	186	2.58	6.66	7.15	86.96	17.07	10.94
**PW**	WW17	1,599.65	543.50	575.76	3,575.42	27.57	33.64	43.50	59.82	732.57	45.80
	DS17	472.43	256.80	120.45	1,535.34	41.01	53.89	67.72	63.33	356.62	75.49
	WW18	2,421.35	530.42	665.45	3,900.32	13.29	21.93	25.64	73.14	799.14	33.00
	DS18	2,302.02	480.84	345.42	3,395.33	13	20.73	24.47	71.77	711.41	30.90
	WW20	2,726.34	589.07	835.24	4,130.35	11.95	21.65	24.73	76.66	909.55	33.36
	DS20	1,818.87	456.85	865.32	3,195.46	19.35	25.12	31.71	62.75	636.94	35.02
**SL**	WW17	7.87	0.85	5.32	10.33	7.23	10.68	12.89	68.55	1.22	15.56
	DS17	7.81	0.89	6.14	10.33	4.18	17.45	17.94	94.56	2.33	29.86
	WW18	6.85	0.83	5.21	9.53	2.98	11.05	11.45	93.23	1.29	18.78
	DS18	6.69	0.64	5.32	9.50	3.75	8.37	9.17	83.26	0.90	13.44
	WW20	7.10	0.99	5.12	9.32	1.99	12.76	12.91	97.63	1.58	22.18
	DS20	6.44	0.90	4.52	9.39	2.71	12.96	13.24	95.81	1.44	22.32
**SNPS**	WW17	23.53	3.82	19	32	4.55	16.19	16.82	92.68	6.46	27.44
	DS17	24.50	4.08	17	39	2.19	16.65	16.79	98.30	7.12	29.06
	WW18	20.52	4.49	11	31	1.97	21.68	21.77	99.18	7.80	38.00
	DS18	19.73	4.47	11	31	11.15	22.56	25.16	80.38	7.02	35.60
	WW20	23.40	4.93	11	36	2.63	20.82	20.98	98.43	8.51	36.35
	DS20	22.05	4.56	11	39	2.47	20.59	20.74	98.58	7.93	35.97
**SWPP**	WW17	155.71	79.35	20.33	398.45	72.79	51.25	89.02	33.15	80.87	51.93
	DS17	35.24	16.84	12.45	122.38	64.98	47.97	80.77	35.28	17.67	50.15
	WW18	135.47	38.37	50.22	235.25	29.71	28.62	41.25	48.14	47.35	34.95
	DS18	109.01	39.86	33.11	219.42	34.88	36.60	50.56	52.41	50.84	46.63
	WW20	511.78	143.88	125.43	845.30	23.05	27.98	36.25	59.58	194.53	38.01
	DS20	120.03	71.82	30.54	375.64	54.77	59.44	80.83	54.08	92.34	76.93
**THSW**	WW17	2.93	1.07	1.32	6.11	31.29	35.36	47.22	56.09	1.37	46.62
	DS17	2.10	0.85	1.24	4.39	20.19	40.78	45.50	80.32	1.35	64.32
	WW18	3.48	0.68	1.09	4.89	7.79	18.10	19.70	84.38	1.02	29.26
	DS18	2.05	0.65	1.05	3.97	20.30	26.86	33.67	63.64	0.77	37.71
	WW20	4.57	0.57	2.36	6.43	2.60	10.20	10.53	93.89	0.79	17.40
	DS20	4.32	0.59	2.15	7.42	2.81	10.80	11.16	93.67	0.79	18.40
**HI**	WW17	10.91	6.55	1.32	32.34	51.86	9.5	52.73	3.24	0.33	3.01
	DS17	8.53	3.43	3.76	21.46	50.98	39.84	64.70	37.91	3.68	43.17
	WW18	5.78	1.75	3.31	13.17	34.31	30.09	45.63	43.48	2.02	34.92
	DS18	4.88	1.87	2.70	15.75	40.95	37.74	55.69	45.92	2.20	45.01
	WW20	18.73	3.33	7.45	27.33	21.39	17.64	27.72	40.48	3.70	19.75
	DS20	6.39	2.70	2.45	16.35	46.58	41.3	62.26	44.01	3.08	48.22
**SY**	WW17	2,594.18	1,096.08	396.34	7,666.34	25.77	42.10	49.36	72.74	1,639.3	63.19
	DS17	1,918.77	986.37	630.71	7,378.25	45.85	57.09	73.22	60.79	1503.2	78.34
	WW18	1,352.23	383.93	500.22	2,350.21	24.32	28.67	37.60	58.16	520.46	38.49
	DS18	1,088.11	398.32	330.32	2,190.25	30.76	36.72	47.90	58.77	539.12	49.55
	WW20	5,125.42	1,443.27	1,250.37	8,450.23	18.61	28.02	33.64	69.41	2,106.05	41.09
	DS20	1,200.34	718.25	300.34	3,750.25	42.13	59.44	72.85	66.55	1,024.36	85.34

DTF, DTSD, DTR, PH, BNPP, PW, SL, SNPS, SWPP, THSW, HI, and SY are the abbreviations of days to flowering, days to silique development, days to ripening, plant height, branch number/plant, plant weight, silique length, seed number/silique, seed weight/plant, thousand seed weight, harvest index, and seed yield, respectively. WW17, DS17, WW18, DS18, WW20, and DS20 are the codes of the two watering regimes during 3 years: well-watered in 2017, drought stress in 2017, well-watered in 2018, drought stress in 2018, well-watered in 2020, and drought stress in 2020. SD, ECV, PCV, GCV, h^2^, GA, and GAM are the abbreviations of standard deviation, environmental coefficient of variation, phenotypic coefficient of variation, genotypic coefficient of variation, heritability in the broad sense, genetic advance, and genetic advance as the percentage of the mean of the studied traits at two watering regimes under 3 years.

The SWPP, SY, HI, and PW traits showed significant differences between normal irrigation and drought stress conditions. Compared with normal irrigation, drought stress significantly reduced SWPP, SY, HI, PW, BNPP, THSW, PH, SL, SNPS, and DTR by 67.08%, 53.62%, 44.09%, 44.09%, 31.92%, 24.58%, 22.85%, 11.51%, 4.03%, 1.73%, and 1.03%, respectively (**Table 1**; **Supplementary Figure S2**).

Analysis of genetic variation showed that the traits were divided into three groups with PCV and GCV above 20% as high, 10%–20% as moderate, and below 10% as low (**Table 1**). In normal irrigation conditions, SWPP, SY, and PW had high PCV and GCV in 3 years (**Table 1**). Moderate PCV and GCV were recorded for SL and SNPS in 3 years. The DTF, DTSD, and DTR traits in 3 years and PH and BNPP in two years showed low PCV and GCVs (**Table 1**). Under drought conditions, the PCV estimates of various characters varied from 0.48% for DTR to 51.08% for SY and SWPP (**Table 1**). The GCV estimates varied from 0.61% for DTR to 80.77% for SWPP. Both GCV and PCV were high (>20%) for PW, SY, SWPP, and HI in 3 years and for SNPS in 2018 and 2020, THSW in 2017 and 2018. DTF, DTSD, and DTR had lower variation (<6%) in drought stress condition in 3 years.

Heritability estimates for most of the traits were higher under drought compared with well-watered conditions. The heritability estimates for traits in the WW condition ranged from 3.24% for HI to 99.18% for SNPS and from 35.28% for SWPP to 99.18% for SNPS in drought stress. The estimates of heritability were moderate for PW and SY in 3 years (**Table 1**). Genetic advances (GA) ranged from 0.33 for HI to 2106.05 for SY in normal irrigation conditions, and it ranged from 0.77 for THSW to 1503.2 for SY in drought stress. High heritability values coupled with high GA were recorded for PW, SWPP, and SY under well-watered conditions in three growing seasons. Genetic advance normalized based on the trait mean (GAM) under well-watered conditions ranged from 63.19% for SY to 0.75% for DTR and from 0.65% for DTR to 85.34% for SY in drought stress. Among the tested traits, SNPS showed high heritability (>80%) coupled with the high GAM (>20%) under both irrigation conditions across 3 years.

### 3.2 Interrelationship of agronomic and yield-related traits

Analysis of correlation of traits helps in indirect selection for yield improvement. Under WW conditions, significant and relatively high correlations were identified for DTF with DTSD and DTR (0.85**, 0.60**) and DTR with DTSD (0.61**) (**Figure 1N**). Correlations of SY with PW (0.62**) and SWPP (0.83**) were significant. Under drought conditions, a positive and significant correlation coefficient was found for SWPP with each SY, HI, and PW traits (0.65**, 0.60**, and 0.54**) whereas DTSD had a strong positive correlation (0.95**) with DTF (**Figure 1O**).

### 3.3 Morphological variations between high- and low-yield varieties

**Figure 2:**
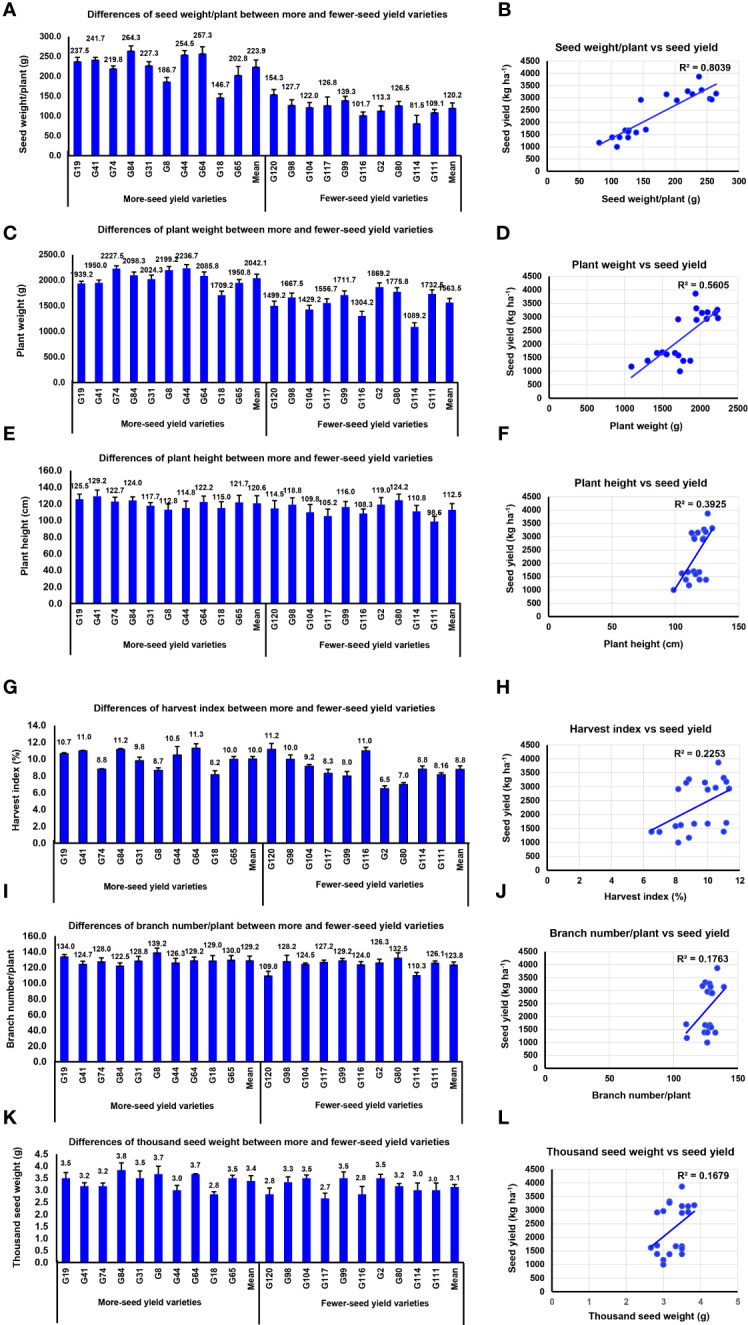
The seed yield variation was caused by agronomic and yield components. **(A)** Differences of seed weight/plant between high- and low-yield varieties. **(B)** Correlation analysis between seed yield and seed weight/plant. **(C)** Differences of plant weight between high- and low-yield varieties. **(D)** Correlation analysis between seed yield and plant weight. **(E)** Differences of plant height between high- and low-yield varieties. **(F)** Correlation analysis between seed yield and plant height. **(G)** Differences of harvest index between high- and low-yield varieties. **(H)** Correlation analysis between seed yield and harvest index. **(I)** Differences of branch number/plant between high- and low-yield varieties. **(J)** Correlation analysis between seed yield and branch number/plant. **(K)** Differences of thousand seed weight between high- and low-yield varieties. **(L)** Correlation analysis between seed yield and thousand seed weight.

Owing to the lower complexity of yield components and lower influence of environmental effects compared with yield, use of yield-related traits with high heritability as indirect selection for improvement of grain yield is preferred. We assessed the difference of agronomic traits and yield components in the high- and low-grain yield varieties. The results indicated that BNPP, SWPP, and PW were significantly larger in the high-yield varieties than in the low-yield varieties (*P*-value < 0.01**). However, PH, THSW, and HI were relatively similar between the two contrasting groups (**Figures 2A–L**).

### 3.4 Distribution of SNPs, LD, LD decay, and population structure

After filtering low-quality SNPs (call rate <90% and minor allele frequency <0.05) in TASSEL software, a set of 29,310 high-quality SNPs was used for genetic variation and GWAS analyses. The SNP markers were not evenly distributed across the whole genome with the A subgenome having a higher number of SNP markers (14,925; 50.92%) compared to the C subgenomes (**Supplementary Figures S3A, D**). However, the density of SNPs in the C subgenome (42.96 SNPs/kbps) was higher than that in the A subgenome (19.76 SNPs/kbps) (**Additional File 4**). Among all chromosomes, C04 (2,624 SNPs) and C05 (723 SNPs) had the highest and lowest numbers of markers (**Supplementary Figure S3A**). The PIC values for chromosome ranged from 0.24 to 0.38 (**Additional File 4**). The mean PIC values of the A and C subgenomes were 0.32 and 0.32, respectively.

Linkage disequilibrium was examined as the squared Pearson correlation coefficient (*r^2^*) between all pairs of SNP markers. The LD in the C subgenome was significantly higher LD (0.031) than in the A subgenome (0.025). The C05 chromosome showed the highest LD (0.088) among chromosomes (**Additional File 4**). The LD decay with an average of 288 kbps in the whole genome ranged from 101.61 in A10 to 953.78 kbps in A08. However, the LD decay distance for C03 was 942.6 kbps, which was large compared with those for other chromosomes in the C subgenome (**Additional File 4**).

In analysis of population structure, the peak of the broken line was observed at k = 7 suggesting that the tested population can be divided into seven distinct groups and one mixed group partly correlated with their origins (**Supplementary Figure S3B**). Neighbor-joining (NJ) cluster analysis was performed to explore the relatedness among the rapeseed varieties. The NJ tree showed that our varieties could be divided into four groups (**Supplementary Figure S3C**). The first group (group A) was composed of 30 breeding lines originated from Iran and Australia, group B was composed of 10 varieties from Iran and Germany, 55 breeding lines in group C originated from Iran, and Group D had 24 varieties composed of hybrids and cultivars originated in France and America. The PCA based on the genome-wide SNPs supported the results of population structure and phylogenetic tree (**Supplementary Figure S3E**).

### 3.5 Significant SNPs and candidate genes associated with traits

We measured 12 traits including plant architecture, phenological and agronomic traits, and yield components in 119 rapeseed varieties grown under two irrigation conditions over 3 years. Using 29,310 high-quality SNPs, our GWAS for these 12 traits revealed 1,281 unique SNPs linked with the traits at the *P*-value < 10^−4^ threshold (**Supplementary Figures S3F**, **S4**-**S6**; **Additional Files 5**-**8**). Higher numbers of significant SNPs associated with traits were identified in the GLM + Q and MLM + K + PC models. However, the result of the MLM model was preferred, which showed fewer false positives than the GLM model. False positives are often controlled by incorporating covariates for the kinship and PCs matrices in the MLM model (Liu et al., 2016). Accordingly, the results of the MLM + K + PC model were used for further analysis. The number of significant SNPs linked with traits was variable. Higher SNPs were found for the DTF, BNPP, and SY traits than others. Based on statistical significance and the repeatability of the linked SNPs in two irrigation treatments across years, several important SNPs are shown in **Tables 2**, **3**. Generally, the effective candidate regions with significant GWAS signals were defined as the LD blocks surrounding the signal peak (Yano et al., 2016). Based on the 288-kbps LD decay distance and CG analysis, we identified 2,232 genes as GWAS-associated CGs (**Additional Files 9**, **10**). The SNPS (557) trait followed by DTF (524) showed the highest number of CGs. The key genes related to four agronomically important traits were selected for further functional verifications.

**Table 2:** Repetitive significant SNP in the current study.

				2017		2018		2020	
Traits	SNP name	Chr	Position	WW	DS	WW	DS	WW	DS
DTF	Bn-A04-p1865434	A04	1588506	ns	4.67E-04	ns	9.61E-04	ns	ns
	Bn-A04-p2568394	A04	2279208	ns	3.62E-04	ns	8.59E-05	ns	ns
	Bn-A10-p15330596	A10	16164284	ns	4.46E-04	ns	9.33E-04	ns	ns
	Bn-A10-p15361519	A10	16139771	ns	2.15E-05	ns	8.88E-04	ns	ns
	Bn-A10-p15405149	A10	16091976	ns	1.37E-04	ns	4.87E-04	ns	ns
	Bn-A10-p15542820	A10	222927	1.77E-04	2.80E-04	ns	9.52E-04	ns	ns
	Bn-A10-p7252424	A10	8893827	ns	4.81E-04	ns	6.67E-05	ns	ns
	Bn-A10-p7347530	A10	8998128	ns	5.02E-04	ns	1.94E-04	ns	ns
	Bn-scaff_22728_1-p744551	C03	5904544	ns	9.02E-04	ns	2.97E-04	ns	ns
THSW	Bn-scaff_15818_1-p427676	C06	15724425	ns	ns	ns	4.40E-04	1.60E-04	2.40E-04
	Bn-scaff_15818_1-p453625	C06	15751833	ns	ns	ns	4.40E-04	1.60E-04	2.40E-04
	Bn-scaff_15818_1-p469375	C06	15766868	ns	ns	ns	4.40E-04	1.60E-04	2.40E-04
	Bn-scaff_15818_1-p471106	C06	15768599	ns	ns	ns	4.40E-04	1.60E-04	2.40E-04
	Bn-scaff_18702_1-p263991	C02	16251517	ns	ns	ns	5.53E-04	7.18E-04	9.53E-04
	Bn-scaff_18702_1-p270197	C02	16260356	ns	ns	ns	5.53E-04	7.18E-04	9.53E-04
	Bn-scaff_18702_1-p288731	C02	16279001	ns	ns	ns	5.53E-04	7.18E-04	9.53E-04
	Bn-scaff_18702_1-p323368	C02	16314729	ns	ns	ns	5.53E-04	7.18E-04	9.53E-04
	Bn-scaff_18702_1-p361232	C02	16355013	ns	ns	ns	5.53E-04	7.18E-04	9.53E-04
	Bn-scaff_18702_1-p365839	C02	16359619	ns	ns	ns	5.53E-04	7.18E-04	9.53E-04

DTF and THSW are the abbreviations of days to flowering and thousand seed weight. WW and DS are the codes of the two watering regimes; well-watered and drought stress during 3 years: 2017, 2017, and 2020.

**Table 3:** Details of 49 pleiotropic SNPs of agronomic and yield-related traits detected from genome-wide association study (GWAS).

SNP name	Chr	Allele			Number			Phenotype			Traits	Near locus previously reported in the same chromosome
Bn-A02-p8191099	A02	AA	AC	CC	15	20	83	186.0	182.3	183.0	DTF	New
								193.8	189.4	190.7	DTSD	
Bn-A02-p8284992	A02	AA	AG	GG	15	18	84	186.0	181.5	183.2	DTF	New
								193.8	189.1	190.8	DTSD	
Bn-A02-p8323616	A02	AA	AC	CC	16	19	84	185.8	181.8	183.1	DTF	New
								193.5	189.0	190.8	DTSD	
Bn-A02-p8440451	A02	AA	AG	GG	24	31	54	185.1	181.3	183.5	DTF	New
								192.7	188.9	191.1	DTSD	
Bn-A02-p8660632	A02	AA	AG	GG	18	25	76	185.4	182.1	184.4	DTF	*BnaA02g12130D*, *BnaA02g12260D* (Zheng et al., 2017)
								186.8	189.5	191.6	DTSD	
Bn-A02-p8934537	A02	AA	AG	GG	18	20	81	185.2	182.5	183.0	DTF	Bn-A02-p3539297 (Xu et al., 2015)
								193.1	190.0	190.6	DTSD	
Bn-A02-p8999771	A02	AA	AC	CC	19	21	79	185.1	182.4	183.0	DTF	Bn-A02-p3539297 (Xu et al., 2015)
								192.9	189.8	190.6	DTSD	
Bn-A02-p9000921	A02	AA	AG	GG	79	21	19	183.0	182.4	185.1	DTF	Bn-A02-p3539297 (Xu et al., 2015)
								190.6	189.8	192.9	DTSD	
Bn-A02-p8190375	A02	AA	AG	GG	15	20	83	168.0	182.3	183.0	DTF	New
								193.8	189.4	190.7	DTSD	
Bn-A10-p13390065	A10	AA	AG	GG	6	14	98	179.5	178.96	184.0	DTF	*BnaA10g18420D*, *BnaA10g18480D, BnaA10g22080D, BnaA10g24300D* (Helal et al., 2021)
								187.3	187.26	191.5	DTSD	
Bn-A10-p15668415	A10	AA	AG	GG	12	17	89	179.8	180.5	184.1	DTF	New
								187.6	188.7	191.7	DTSD	
Bn-A01-p17377721	A01	AA	AC	CC	4	25	88	187.0	190.7	191.2	DTSD	New
								121.0	116.2	118.2	PH	
Bn-A01-p27079797	A01	AA	AG	GG	33	83	2	192.0	190.4	191.9	DTSD	New
								117.8	118.1	121.3	PH	
Bn-A01-p21758046	A01	AA	AG	GG	51	42	25	191.2	190.4	191.0	DTSD	*BnaA01g26410D*-*BnaA01g26530D* (Lu et al., 2017)
								118.3	117.6	117.7	PH	
								9.5	8.7	9.1	HI	
Bn-A01-p5715141	A01	AA	AG	GG	27	28	62	191.9	190.7	190.5	DTSD	*Bna.QRT3* (*BnaA01g10390D*) (Lu et al., 2017)
								118.4	117.5	117.7	PH	
								9.3	8.8	9.1	HI	
Bn-A01-p6678914	A01	AA	AG	GG	93	16	6	266.8	266.5	266.3	DTR	Bn-A01-p7430311 (Sun et al., 2016b)
								117.8	116.8	118.2	PH	
Bn-A01-p9203096	A01	AA	AG	GG	8	19	90	265.6	266.1	266.8	DTR	New
								118.1	117.8	117.9	PH	
Bn-scaff_18936_1-p102755	C03	AA	AG	GG	75	27	13	266.8	266.5	266.4	DTR	New
								118.2	116.6	117.1	PH	
Bn-scaff_18936_1-p240670	C03	AA	AG	GG	16	29	72	266.4	266.4	266.8	DTR	New
								116.9	117.4	118.3	PH	
Bn-scaff_18936_1-p269153	C03	AA	AC	CC	95	19	4	266.7	266.4	267.7	DTR	New
								118.3	116.6	113.7	PH	
Bn-scaff_18936_1-p472353	C03	AA	AG	GG	17	36	65	266.9	266.4	266.7	DTR	New
								116.8	118.2	118.0	PH	
Bn-scaff_18936_1-p93643	C03	AA	AG	GG	13	27	76	266.4	266.5	266.8	DTR	New
								117.1	117.0	118.2	PH	
Bn-scaff_18936_1-p97644	C03	AA	AC	CC	76	28	13	266.8	266.5	266.4	DTR	New
								118.2	117.0	117.1	PH	
Bn-scaff_18936_1-p274133	C03	AA	AG	GG	5	17	95	267.3	266.4	266.7	DTR	New
								114.4	116.5	118.3	PH	
Bn-A01-p7619726	A01	AA	AG	GG	102	12	5	265.6	265.7	266.8	DTR	New
								117.8	117.0	122.0	PH	
								9.1	9.0	9.8	HI	
Bn-A01-p8014995	A01	AA	AG	GG	4	9	104	266.9	265.6	266.7	DTR	BnvaA0107152286 (Han et al., 2022)
								118.7	115.6	118.0	PH	
								9.1	8.7	9.2	HI	
Bn-scaff_18936_1-p439378	C03	AA	AG	GG	5	17	96	267.3	265.8	266.8	DTR	New
								114.4	117.1	118.2	PH	
								1,762.8	1,875.1	1,905.8	PW	
Bn-scaff_18936_1-p440619	C03	AA	AG	GG	5	17	96	266.8	265.8	267.3	DTR	New
								114.4	117.1	118.2	PH	
								1,762.8	1,875.1	1,905.8	PW	
Bn-scaff_18936_1-p559490	C03	AA	AC	CC	3	20	95	267.4	265.9	266.8	DTR	New
								112.8	116.3	118.4	PH	
								1,539.0	1,878.7	1908.4	PW	
Bn-scaff_18936_1-p610540	C03	AA	AG	GG	94	20	4	267.8	266.1	266.7	DTR	New
								118.4	116.2	114.4	PH	
								1,911.3	1,867.0	1662.0	PW	
Bn-scaff_18936_1-p611810	C03	AA	AC	CC	94	20	4	267.8	266.1	266.7	DTR	New
								118.4	116.2	114.4	PH	
								1,911.3	1,867.0	1662.0	PW	
Bn-scaff_18936_1-p618378	C03	AA	AG	GG	4	20	94	267.8	266.1	266.7	DTR	*BnaC03g45540D* (Zhang et al., 2023a)
								114.4	116.2	118.4	PH	
								1,662.0	1,867.0	1911.3	PW	
Bn-scaff_18936_1-p622547	C03	AA	AC	CC	94	20	4	266.7	266.1	267.8	DTR	New
								118.4	116.2	114.4	PH	
								1,911.3	1,867.0	1662.0	PW	
Bn-scaff_18936_1-p643990	C03	AA	AG	GG	94	20	4	266.7	266.1	267.8	DTR	New
								118.4	116.2	114.4	PH	
								1,911.3	1,867.0	1662.0	PW	
Bn-A08-p12555227	A08	AA	AG	GG	11	20	86	1,936.4	1,903.4	1882.5	PW	New
								197.6	177.1	174.9	SWPP	
								9.9	9.0	9.0	HI	
								2,507.1	2,172.5	2196.8	SY	
Bn-A08-p15782077	A08	AA	AG	GG	70	29	20	1,822.2	1954.1	2041.1	PW	New
								168.1	182.7	204.7	SWPP	
								9.1	9.0	9.5	HI	
								2,127.5	2,255.8	2520.0	SY	
Bn-A08-p15782229	A08	AA	AG	GG	70	28	20	1,822.2	1,947.9	2041.1	PW	New
								168.1	182.1	204.7	SWPP	
								9.1	9.0	9.5	HI	
								2,127.5	2,234.1	2520.0	SY	
Bn-A08-p12556455	A08	AA	AG	GG	88	10	11	1,887.5	1,832.9	2098.3	PW	New
								175.0	179.7	264.3	SWPP	
								2,201.0	2,151.8	3177.8	SY	
Bn-A08-p13626189	A08	AA	AG	GG	72	31	15	1,890.8	1,870.0	1932.4	PW	New
								175.3	175.7	189.7	SWPP	
								2,191.0	2,198.2	2368.3	SY	
Bn-A08-p13626982	A08	AA	AG	GG	16	32	70	1,906.0	1,895.2	1885.0	PW	New
								188.7	175.6	175.4	SWPP	
								2,339.1	2,190.0	2198.8	SY	
Bn-A08-p13638847	A08	AA	AC	CC	69	33	16	1,889.1	1,886.3	1906.0	PW	New
								175.8	174.8	188.7	SWPP	
								2,201.5	2,184.7	2339.1	SY	
Bn-A08-p13670107	A08	AA	AG	GG	15	28	72	1,932.4	1,880.5	1890.8	PW	New
								189.7	176.0	175.3	SWPP	
								2,368.3	2,217.4	2191.0	SY	
Bn-A08-p14538807	A08	AA	AG	GG	87	19	8	1,873.6	1,955.0	1930.9	PW	New
								171.6	193.4	185.4	SWPP	
								2,163.2	2,330.7	2371.0	SY	
Bn-A08-p15994149	A08	AA	AC	CC	29	25	63	1,988.2	1,901.6	1839.1	PW	*Bna.BBX20* (*BnaA08g16780D*, *AT4G39070*) (Lu et al., 2017) *Bna.BBX15* (*BnaA08g19420D*) (Lu et al., 2017)
								198.4	178.7	167.3	SWPP	
								2,432.6	2,196.9	2132.8	SY	
Bn-scaff_19208_1-p78898	C04	AA	AG	GG	66	32	20	1,883.9	1,828.8	2002.8	PW	New
								178.4	161.4	200.9	SWPP	
								2,196.2	2,098.2	2489.5	SY	
Bn-scaff_19208_1-p82535	C04	AA	AG	GG	66	31	20	1,883.9	1,827.1	2002.8	PW	New
								178.4	161.5	200.9	SWPP	
								2,196.2	2,103.9	2489.5	SY	
Bn-scaff_19208_1-p93814	C04	AA	AC	CC	66	31	21	1,883.9	1,827.1	2002.8	PW	New
								178.4	161.5	200.9	SWPP	
								2,196.2	2,103.9	2489.5	SY	
Bn-scaff_19208_1-p94498	C04	AA	AC	CC	66	32	21	1,883.9	1,828.8	2002.8	PW	New
								178.4	161.4	200.9	SWPP	
								2,196.2	2,098.2	2489.5	SY	
Bn-scaff_19208_1-p94501	C04	AA	AG	GG	20	31	66	2,002.8	1,827.1	1883.9	PW	New
								200.9	161.5	178.4	SWPP	
								2,489.5	2,103.9	2196.2	SY	

DTF, DTSD, DTR, PH, PW, SWPP, HI, and SY are the abbreviations of days to flowering, days to silique development, days to ripening, plant height, plant weight, seed weight/plant, harvest index, and seed yield, respectively.

#### 3.5.1 SNPs and candidate genes linked with phenological and agronomic traits under drought conditions

##### 3.5.1.1 Days to flowering

**Figure 3:**
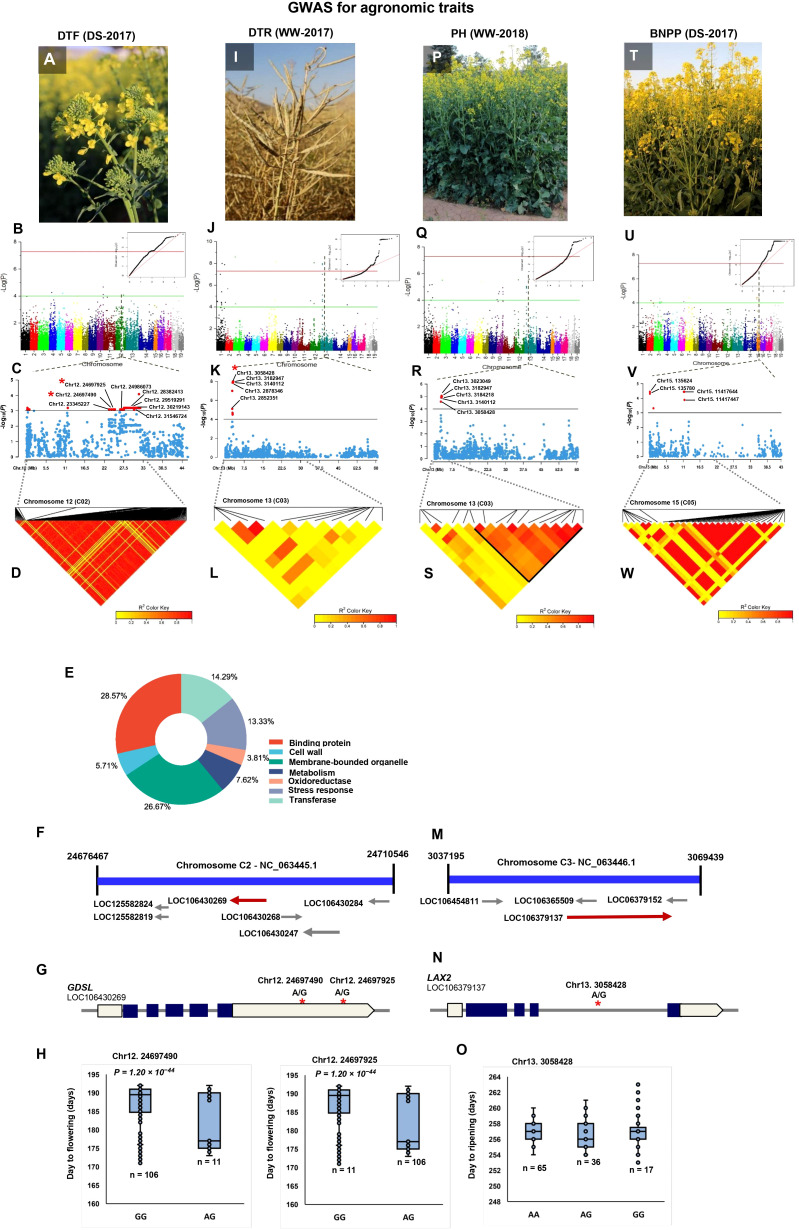
Genome-wide association study (GWAS) reveals the genetic basis of agronomic traits. **(A)** GWAS of day to flowering (DTF) using the 119 rapeseed (*Brassica napus*) varieties under drought stress conditions in 2017. **(B)** Manhattan plot and corresponding quantile–quantile (QQ) plot displaying the GWAS result of DTF in 19 chromosomes (1–10 stand for rapeseed chromosome of A01–A10, and 11–19 stand for rapeseed chromosome of C01–C09 at the horizontal axis). SNPs on different chromosomes are denoted by different colors. **(C)** Locus zoom plot for DTF associations in chromosome 12 (C02). **(D)** A representation of pairwise *r* ^2^ value (displayed as percentages) among polymorphic sites of chromosome 12 (C02) for DTF. **(E)** CirGO visualization of GO enrichment analysis of significant genes identified by GWAS for DTF. **(F)** Schematic diagram of the region of chromosome 12 (C02) genotyped in this study, showing the associated gene *GDSL* (LOC106430269). Chromosomal position based on the National Center for Biotechnology Information (NCBI). **(G)** Gene model of *GDSL*. Solid boxes indicate exons, open boxes indicate untranslated regions (UTRs), and lines connecting the exons indicate introns. The red stars mark the position of Chr12:24697490 and Chr12:24697925. **(H)** The influence of Chr12:24697490 and Chr12:24697925 on day to flowering. The significance of difference between two varieties was evaluated using Student’s t-test. **(I)** GWAS of day to ripening (DTR) under well-watered conditions in 2017. **(J)** Manhattan plot and corresponding quantile–quantile (QQ) plot displaying the GWAS result of DTR in 19 chromosomes. **(K)** Locus zoom plot for DTR associations in the chromosome 13 (C03). **(L)** A representation of pairwise *r* ^2^ value (displayed as percentages) among polymorphic sites of chromosome 13 (C03) for DTR. **(M)** Schematic diagram of the region of chromosome 13 (C03) genotyped in this study, showing the associated gene *LAX2* (LOC106379137). Chromosomal position based on NCBI. **(N)** Gene model of *LAX2*. Solid boxes indicate exons, open boxes indicate untranslated regions (UTRs), and lines connecting the exons indicate introns. The red star marks the position of Chr13:3058428. **(O)** The influence of Chr13:3058428 on day to ripening. **(P)** GWAS of plant height (PH) under well-watered conditions in 2018. **(Q)** Manhattan plot and corresponding quantile–quantile (QQ) plot displaying the GWAS result of PH in 19 chromosomes. **(R)** Locus zoom plot for PH associations in the chromosome 13 (C03). **(S)** A representation of pairwise *r* ^2^ value (displayed as percentages) among polymorphic sites of chromosome 13 (C03) for PH. **(T)** GWAS of branch number/plant (BNPP) under drought stress conditions in 2017. **(U)** Manhattan plot and corresponding quantile–quantile (QQ) plot displaying the GWAS result of BNPP in 19 chromosomes. **(V)** Locus zoom plot for BNPP associations in the chromosome 15 (C05). **(W)** A representation of pairwise *r* ^2^ value (displayed as percentages) among polymorphic sites of chromosome 15 (C05) for BNPP.

A total of 324 significant SNPs were significantly associated with the DTF (**Additional Files 5**-**8**). The SNP Chr10:222927 linked with flowering time in both WW and DS conditions across 2 years (**Table 2**). Six linked SNPs, (Chr10:222927, Chr10:8893827, Chr10:8998128, Chr10:16091976, Chr10:16091976, Chr10:16139771) were significant across 2 years under drought conditions (**Table 2**). We found that the C02 peak was a major associated signal, harboring 52.77% of the associated SNPs (171) (**Additional File 5**; **Figures 3A–D**), which had not been reported previously. Accordingly, we focused on the 159 SNPs positioned in the Chr12:23345227–32168120 genomic region and extracted all CGs within 200 kbps of the most significant SNPs. A number of 146 genes, including transcription factors, enzymes, and transporters that represent plausible candidates for the causal gene of the flowering time, were identified (**Additional File 10**). Information of seven enriched gene ontology groups for DTF candidate genes is shown in **Figure 3E**. The SNP Bn-scaff_18507_1-p354053 (A/G) located 12.7 kbps downstream of *LIPOXYGENASE 4* (*LOX4*) plays important roles in flower development and male fertility regulation (Klepikova et al., 2016). The Chr12:24697490 (A/G) and Chr12:24697925 (A/G) SNPs in a LD block were located within the position of candidate flowering gene *GDSL ESTERASE/LIPASE* (**Figures 3F, G**). Our results showed that the varieties with the alleles GG in SNP Chr12:24697490 showed significantly late flowering compared with those with the alleles AG (**Figure 3H**). The 1-kbps LD block surrounding Chr12:24986073 (A/C) contains the candidate flowering gene *FAR1-RELATED SEQUENCE (FRS).* The 3.2-kbps LD block surrounding Chr12:28382413 (A/G) contains the candidate flowering gene *9-CIS-EPOXTCAROTENOID DIOXYGENASE* (*NCED9*), which is a critical gene in the regulation of abscisic acid (ABA) synthesis. The 60.8-kbps LD block surrounding the Chr12:29519291 (A/G) SNP contains *CINNAMOYL-COA REDUCTASE 2* (*CCR2*), which plays an important role in pollen development by regulating the programmed cell death (PCD) of tapetum cells (Zhang et al., 2023c). Chr12:30219143 (A/G), which explained 11.62% of the phenotypic variance located 3.6 kbps downstream of pollen-specific gene *RALFL14*. Another pollen-specific gene, *DEFENSIN-LIKE 7* (*DEFL7*), is located 36.71 kbps downstream of Chr12:30219143 (*P* = 6.58 × 10^−4^). *DEFL* genes are involved in pollen tube guidance and pollen tube reception and are responsible for the failure of double fertilization events (Takeuchi and Higashiyama, 2012).

##### 3.5.1.2 Days to silique development

GWAS identified 30 TASs on chromosomes A01, A02, A03, and A10 for DTSD (**Additional Files 5**-**8**). Chr1:18351464 and Chr1:5217408 had the strongest signals for DTSD in the WW condition in 2017, which explained 43.50% and 37.75% of the phenotypic variance, respectively (**Additional File 5**). Three SNPs under drought conditions, Chr3:25485861 (A/G), Chr3:25524140 (A/G), and Chr3:25525060 (A/C), identified on chromosome A03 were located ~20 kbps downstream and upstream of the *AGL19* gene, respectively. This gene is involved in seed formation, silique maturity, and seed desiccation (Shah et al., 2022).

##### 3.5.1.3 Days to ripening

A total of 97 significant associations were identified for DTR (**Additional Files 5**-**8**). The 55 and 42 TASs identified in WW and DS conditions explained 63.20% and 36.79% of the phenotypic variance. There were 77 CGs associated with 21 significant SNPs in the C03:2498421–3217123 intervals (**Additional Files 5**, **10**; **Figures 3I–L**). Chr13:2852351 (*P* = 2.11 × 10^−5^) and Chr13:2878346 (*P* =1.36 × 10^−8^) were located ~10 kbps downstream and upstream of aspartic proteinase oryzasin-1, respectively. In *B*. *napus*, *BnaAP36s* and *BnaAP39s* genes play a critical role in pollen tube growth (Wang et al., 2023). Chr13:3058428 (*P* =1.36 × 10^−8^) explained 42.99% of the phenotypic variance located within the position of the panicle architecture-related gene *LAX PANICLE 2* (*LAX2*) (**Figures 3M, N**). The varieties with the AA and GG alleles in this SNP showed significantly late maturity compared with those with the AG alleles (**Figure 3O**). Another specific gene, *FY*, located 19.96 kbps upstream of the Chr13:3140112 (*P* = 1.07 × 10^−8^) SNP, plays a role in the regulation of flowering time in the autonomous flowering pathway through repression of the FLOWERING LOCUS C (FLC) (Kyung et al., 2022). Chr13:3182947 which explained 43.65% of the phenotypic variance was located <1 kbps upstream of two genes from MADS-box *AGAMOUS* (*AG*) genes: *AGL15* and *AGL16*. MADS-box genes play an important role in regulating floral carpel and ovule development (Sheng et al., 2019; Shah et al., 2022).

##### 3.5.1.4 Plant height

A total of 70 significantly associated SNPs were identified for PH, of which 59 and 11 were identified in the WW and DS conditions, respectively (**Additional Files 5**-**8**). Among the linked SNPs, 16 SNPs identified in the WW conditions were located on chromosome C03 (2736068–3217123 bp), which explained 31.69% of the phenotypic variance (**Additional File 6**; **Figures 3P–S**). The 14.28-kbps LD block surrounding Chr13:3023049 (*P* = 8.96 × 10^−6^) contains the *TIFY9* gene (**Figure 3R**). The *TIFY* family is a plant-specific gene involved in accelerated cell division, leaf flatness, and lateral organ development (Zhang et al., 2020). Chr13:3058428 (A/G) which explained 27.34% of the phenotypic variance of PH was located 12.78 kbps downstream of gene *GH3.12* responsible for plant stem growth. Three SNPs, Chr13:3140112 (A/G), and Chr13:3182947 (A/C), and Chr13:3184218 (A/G), were located ~20 kbps downstream and upstream of *PAO1*.

#### 3.5.2 SNPs and candidate genes linked with yield and yield-related traits under drought conditions

##### 3.5.2.1 Branch number/plant

Of 250 SNPs detected for BNPP, 28 and 222 SNPs explained 10.58% and 89.14% of the BNPP variance in the WW and DS conditions, respectively (**Additional Files 5**-**8**). There were 22 of the 222 SNPs identified under DS condition located in the C05:11200372–1417644-bp region, which had not been reported previously (**Additional Files 5**, **10**; **Figures 3T–W**). Five SNPs, namely, Chr15:125848, Chr15:135624, Chr15:135780, Chr15:136834, and Chr15:136975, within an LD block were located <5 kbps downstream and upstream of the *SBT1.1* gene (**Figure 3V**), a gene which contributed to elongation of the main shoot, increasing inflorescence branching and biomass (Martinez et al., 2015). Two SNPs, Chr15: 136834 (*P* = 1.31 × 10^−4^) and Chr15: 136975 (*P* = 3.63 × 10^−5^), in an LD block were mapped to 11 kbps upstream of a member of *SKP1-Like* gene family, *ASK3.*

##### 3.5.2.2 Plant weight

**Figure 4:**
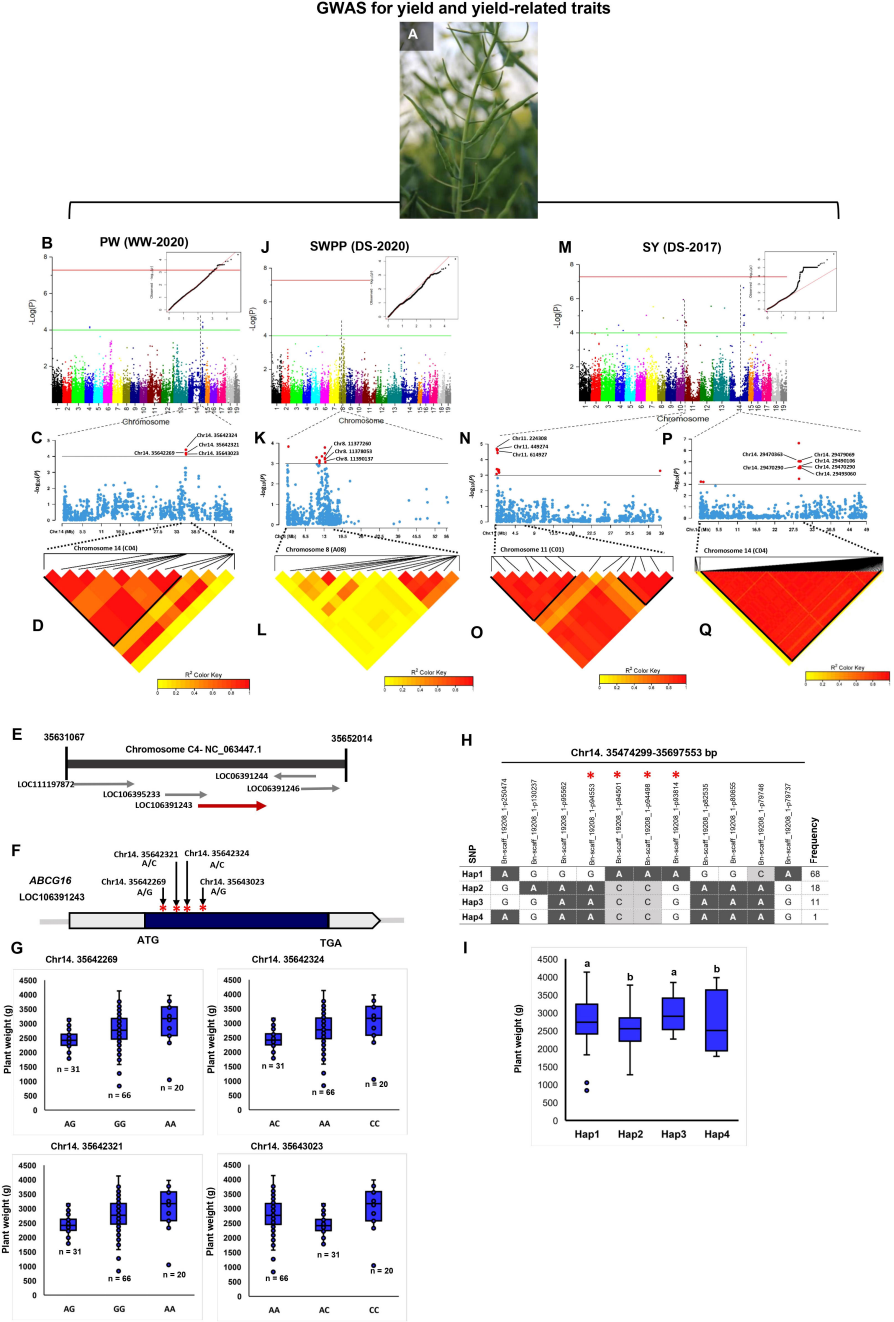
Genome-wide association study (GWAS) reveals the genetic basis of seed yield and yield-related traits in rapeseed (*Brassica napus*). **(A)** Phenotypes of siliques in rapeseed which are considered the major contributing factors for increasing rapeseed yield. **(B–D)** GWAS of plant weight (PW) using the 119 rapeseed varieties under well-watered conditions in 2020. **(B)** Manhattan plot and corresponding quantile–quantile (QQ) plot displaying the GWAS result of PW in 19 chromosomes (1–10 stand for rapeseed chromosomes of A01–A10, and 11–19 stand for rapeseed chromosomes of C01–C09 at the horizontal axis). SNPs on different chromosomes are denoted by different colors. **(C)** Locus zoom plot for PW associations in the chromosome 14 (C04). **(D)** A representation of pairwise *r* ^2^ value (displayed as percentages) among polymorphic sites of chromosome 14 (C04) for PW. **(E)** Schematic diagram of the region of chromosome 14 (C04) genotyped in this study, showing the associated gene *ABCG16* (LOC106391243). Chromosomal position based on the National Center for Biotechnology Information (NCBI). **(F)** Gene model of *ABCG16*. Solid boxes indicate exons, and open boxes indicate untranslated regions (UTRs). The red stars mark the positions of Chr14:35642269, Chr14:35642321, Chr14:35642324, and Chr14:35643023. **(G)** The influence of Chr14:35642269, Chr14:35642321, Chr14:35642324, and Chr14:35643023 on plant weight. **(H)** Haplotype block based on 11 significant SNPs on chromosome 14 (C04). **(I)** Four different haplotype variants (Hap1–Hap4) are present at different frequencies in the analyzed population. Boxplots for plant weight indicate the phenotype values corresponding to the four different haplotype groups. Significant differences among haplotypes were identified using one-way ANOVA. Different letters indicate distinct groups. **(J–L)** GWAS of seed weight/plant (SWPP) under drought stress conditions in 2020. **(J)** Manhattan plot and corresponding quantile–quantile (QQ) plot displaying the GWAS result of SWPP in 19 chromosomes. **(K)** Locus zoom plot for SWPP associations in chromosome 8 (A08). **(L)** A representation of the pairwise *r* ^2^ value (displayed as percentages) among polymorphic sites of chromosome 8 (A08) for SWPP. **(M-Q)** GWAS of seed yield (SY) under drought stress conditions in 2017. (**M**) Manhattan plot and corresponding quantile–quantile (QQ) plot displaying the GWAS result of SY in 19 chromosomes. **(N)** Locus zoom plot for SY associations in chromosome 11 (C01). **(O)** A representation of pairwise *r* ^2^ value (displayed as percentages) among polymorphic sites of chromosome 11 (C01) for SY. **(P)** Locus zoom plot for SY associations in the chromosome 14 (C04). **(Q)** A representation of pairwise *r* ^2^ value (displayed as percentages) among polymorphic sites of chromosome 14 (C04) for SY.

Of the 56 significant SNPs for PW, 34 and 22 explained 58.10% and 41.89% of PW variance in the WW and DS conditions, respectively (**Additional Files 5**-**8**). There were 12 TASs of the WW condition located on the 35,474,299–35,698,370 bp interval in chromosome C04 (**Additional File 7**; **Figures 4A–D**). This genomic region contained four genes of ABC transporter G superfamily (*ABCG16*, *ABCG17*, *ABCG18*, and *ABCG19*) which contribute to cytokinin transport in the shoot and enhance the tiller number, grain number per panicle, and grain yield (Wu et al., 2022a). Four SNPs, Chr14:35642269 (A/G), Chr14:35642321 (A/C), Chr14:35642324 (A/C), and Chr14:35643023 (A/G), in an LD block, were located within the *ABCG16* sequence (**Figures 4C, E, F**). The varieties with the alleles AA and CC in these SNPs showed significantly higher plant weight compared with varieties with other alleles (**Figure 4G**). We identified four haplotypes/markers associated with PW located on chromosome C04 (35,474,299–35,697,553 bp) (**Figure 4H**) with an average plant weight of 2984.37 g in Hap3 significantly greater than in other three Haps (**Figure 4I**).

##### 3.5.2.3 Seed number/silique

There were 10 drought-related TASs identified on chromosome A02 (750,194–791,056 bp) that had not been reported in drought stress in rapeseed, previously (**Additional File 7**). The Chr2:751843 (A/G), Chr2:751923 (A/G), and Chr2:752016 (A/G) SNPs located within a MADS-box gene, *FLOWERING LOCUS C* (*FLC*), involved in flowering time control, inflorescence architecture, floral organ identity determination, and seed development (Soppe et al., 2021). Chr2:989812 (A/C) located 9.10 kbps downstream of seed plant-specific *BIG GRAIN LIKE* gene family (*BG1-D*) regulates grain number per plant, grain size with both bigger length and width, and finally grain yield (Liu et al., 2015; Lo et al., 2020; Gao et al., 2022). Chr7:36944365 (A/C) located within *CAND1* encodes cullin-associated Nedd8-dissociated protein 1 (**Additional File 7**).

##### 3.5.2.4 Seed weight/plant

Of the significant TASs identified in drought stress, 10 were located on 10,335,313 bp–13,452,346 bp of chromosome A08 (**Additional Files 7**, **8**). Chr8:11377260 and Chr8:11378053 (*P* = 7 × 10^−4^) were located within the position of a member of the purine permease (PUP)-type transporter gene family, *PUP21*. In addition, Chr8:11390137 (A/G) was located within other members of the PUP-like family gene, *PUP10* (**Figures 4J–L**).

##### 3.5.2.5 Thousand seed weight

Among the THSW-associated SNPs, 10 were stable overs 2 years under drought stress conditions (**Table 2**). There were 13 TASs on chromosome C02 (16,251,517 bp–16,902,605 bp) and eight on chromosome C06 (15,724,425 bp–15,768,599 bp) that explained 26.08% and 19.7% of the THSW variance in the WW and DS treatments, respectively (**Additional File 7**).

##### 3.5.2.6 Seed yield

Of the SY SNPs, 34 were positioned in the 130,644-bp–676,816-bp interval on chromosome C01 (**Figures 4M–O**). Chr11:224308 (*P* = 2.58 × 10^−5^; **Figure 4N**), which explained 24.34% of the phenotypic variance located within the position of the *DRG3* gene belonging to the G-protein family. Chr11:449274 (*P* = 4.56 × 10^−4^; **Figure 4N**), which explained 17.69% of the SY variance located 7.78 kbps downstream of *CYP79B1*. Chr11:614927 (*P* = 4.68 × 10^−4^) was located within the sequence of the *AGAMOUS LIKE21* (*AGL21*) gene, which has been shown to upregulate in siliques and dry seeds in rapeseed (Yu et al., 2017). There were 112 drought-related SY–SNPs located in the 29,030,148-bp–29,607,326-bp interval on chromosome C04 and explained 67.56% of the phenotypic variance of SY (**Additional File 5**; **Figures 4P, Q**). Six SNPs on chromosome C04 with complete LD (*r^2 =^* 1, **Figure 4Q**), namely, Chr14:29470290, Chr14:29470363, Chr14:29479069, Chr14:29490106, Chr14:29492969, and Chr14:29493060, located <13 kbps downstream and upstream of *CYP78A9*, which is another member of the cytochrome P450 superfamily.

##### 3.5.2.7 Harvest index

The 38 SNPs significantly associated with HI contributed to 38.34% and 61.65% of HI variance in the WW and DS conditions, respectively (**Additional Files 5**-**8**). Among the HI SNPs, seven SNPs were located on chromosome C06 (24,703,167 bp–34,271,977 bp) and five on chromosome C08 (28,759,659 bp–30,043,480 bp) in the WW condition in 2017 (**Additional File 5**).

### 3.6 Identification of novel pleiotropic SNPs associated with more than one trait

In the current study, 19 novel repetitive SNPs linked to both DTF and THSW traits were identified on chromosomes A04, A10, C02, C03, and C06. Analysis of SNPs for pairwise traits revealed that 49 SNPs associated with two or more than two traits and the differences in phenotypic values between varieties with two alleles at each of these SNPs were significant. Of the 49 pleiotropic SNPs, 11 SNPs were overlapped with those reported in previous studies only and the rest were unique in this study (**Table 3**). There were 11 SNPs on chromosome A02 and A10 that were associated with both DTF and DTSD simultaneously. A pleiotropic SNP Bn-A02-p8660632 for the phenotype of both DTF and DTSD mapped on chromosome A02 and was 2,186 kbps downstream of the position of the *BnaA02g12130D* and *BnaA02g12260D* genes. These genes affected DTF and PH in *B*. *napus* in the Zheng et al. (2017) study. Pleiotropic SNPs were identified for DTSD and PH; DTSD, PH, and HI; DTR and PH; DTR, PH, and HI; DTR, PH, and PW; PW, SWPP, HI, and SY; and PW, SWPP, and SY (**Table 3**). The position of the pleiotropic SNP Bn-A08-p15994149 for PW, SWPP, and SY on chromosome A08 was 3,734 kbps downstream of the candidate gene *BnaA08g16780D* and was 69 kbps downstream of the region (13,520,923 bp–13,598,303 bp) that affected branch pod number and pod number per plant in the Lu et al. (2016) study.

### 3.7 Estimating the effect of major pleiotropic SNPs on traits

As shown in **Table 3**, varieties with allele AA in the Bn-A01-p21758046 and Bn-A01-p5715141 SNPs showed higher DTSD, PH, and HI. Varieties with the allele GG in Bn-A01-p7619726 had higher DTR, PH, and HI. The allele GG in Bn-scaff_18936_1-p439378 and Bn-scaff_18936_1-p440619 presented higher DTR, PH, and PW. The allele AA in Bn-scaff_18936_1-p610540 and Bn-scaff_18936_1-p611810 increased DTR, PH, and PW. Varieties with the allele AA in Bn-A08-p12555227 showed higher PW, SWPP, HI, and SY, whereas varieties with the allele GG in Bn-A08-p15782077 and Bn-A08-p15782229 had higher PW, SWPP, HI, and SY.

### 3.8 Comparative transcriptome analysis between seed and leaf of low and high grain yield varieties

**Figure 5:**
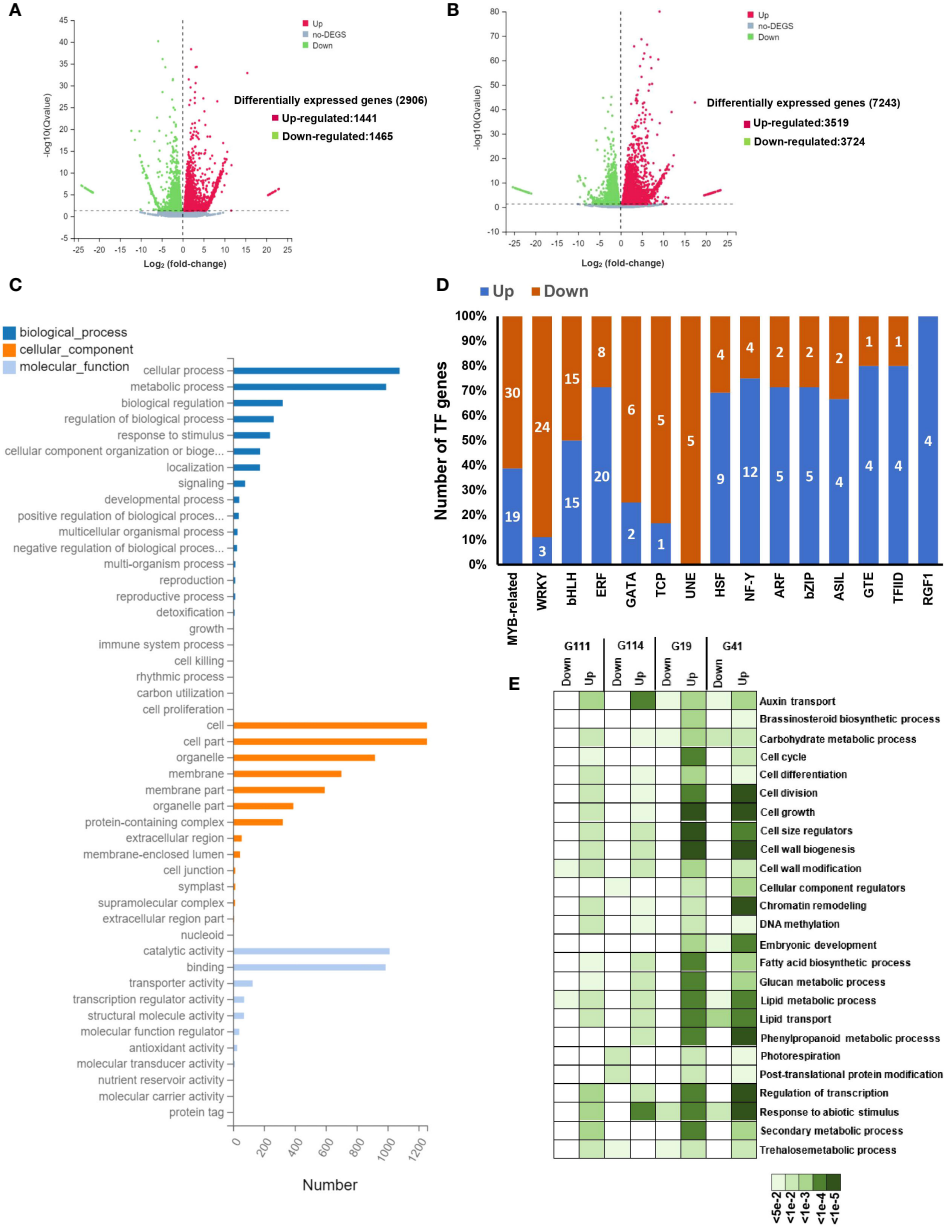
Drought-responsive transcriptional changes in rapeseed (*Brassica napus*). **(A, B)** Volcano plots showing the comparison of differentially expressed genes (DEGs) between the treatment and control groups in low-yield varieties **(A)** and high-yield varieties **(B)**. The red scatters indicate upregulated DEGs, green scatters indicate downregulated DEGs, and black scatters indicate no DEGs between the uniconazole-treated and untreated samples. Datasets were filtered to remove genes with low expression levels (dotted line from −1 to 1 on the x-axis), and a significance cutoff (*P*-value < 0.01) was applied (dotted line on the y-axis). The x-axis shows log2 fold changes in expression and the y-axis –log10 of *q-*values of a transcript being differentially expressed. **(C)** The Gene Ontology (GO) enrichment analysis of differentially expression genes. The top 23 enriched GO terms (*P-*value < 1.0E-5) were used for diagram. Gene ontology categories included biological process, cellular component, and molecular function. **(D)** The number of genes from different transcription factor (TF) families showing up- or downregulation in high-yield varieties of mature seed. **(E)** Enriched gene ontology (GO) terms of mature seed in down- and upregulated genes in high-yield varieties and low-yield varieties. The color scale at the bottom represents significance (corrected *P*-value).

The transcriptome analysis of mature seeds in the rapeseed varieties differing in their seed yield can provide crucial systems-level insights into molecular mechanisms underlying seed development and seed yield. We selected four rapeseed varieties, namely, G111 and G114 as low-seed yield and G19 and G41 as high-seed yield varieties, to investigate the transcriptional differences in the two contrasting groups. In total, 2,906 DEGs (1,441 up- and 1,465 downregulated) of both tissues (seed and leaf) in low-seed yield and 7,243 (3,519 up- and 3,724 downregulated) of both tissues in high-seed yield varieties with |log2FC| ≥ 1 and padj < 0.05 were identified (**Figures 5A, B**). Of the DEGs, 994 upregulated and 1,008 downregulated genes shared between the two contrasting groups. The Gene Ontology (GO) enrichment analyses of all the genes showed that most of the genes were related to various developmental process, reproductive processes, cell wall organization, cell cycle and cell division, metabolic processes, response to stress/hormone, and regulation of transcription (**Figure 5C**). These processes are well known to be involved during various aspects of seed development. At least, 321 transcription factor (TF)-encoding genes belonging to 66 families exhibited stage-specific expression in one or more than one cultivar. The members of MYB, bHLH, ERF, WRKY, bZIP, and ARF families were highly represented in these varieties (**Figure 5D**). The expression profiles of key gene families and individual genes involved in cell division, cell size determination, cell wall modification, carbohydrate metabolism, and grain filling were analyzed. We observed a higher expression of several members of these gene families in high-yield varieties (**Figure 5E**). A higher transcriptional activity of cyclin-encoding genes was identified in high-yield varieties, which is almost related to higher mitotic activity and an extended period of cell division. The genes encoding glucan synthases and xyloglucan endotransglucosylases/hydrolases exhibited higher transcriptional activity in high-yield varieties. These enzymes are involved in the synthesis and remodeling of cell wall components and production of energy (Miedes et al., 2013; Perrot et al., 2022; Zhang et al., 2022). Furthermore, the transcript abundance of genes involved in cell expansion (expansins), seed storage proteins (e.g., vicilin-like storage protein), and lipid transfer proteins was also significantly higher in the high-seed yield rapeseed varieties (**Figure 5E**). It has been shown that these proteins contribute to various aspects of seed development and seed maturation (Pagnussat et al., 2012; Wang et al., 2015a; Yaqoob et al., 2020; Rahman et al., 2021; Fang et al., 2023).

### 3.9 Validation of candidate genes associated with SNPs by transcriptome analysis

**Figure 6:**
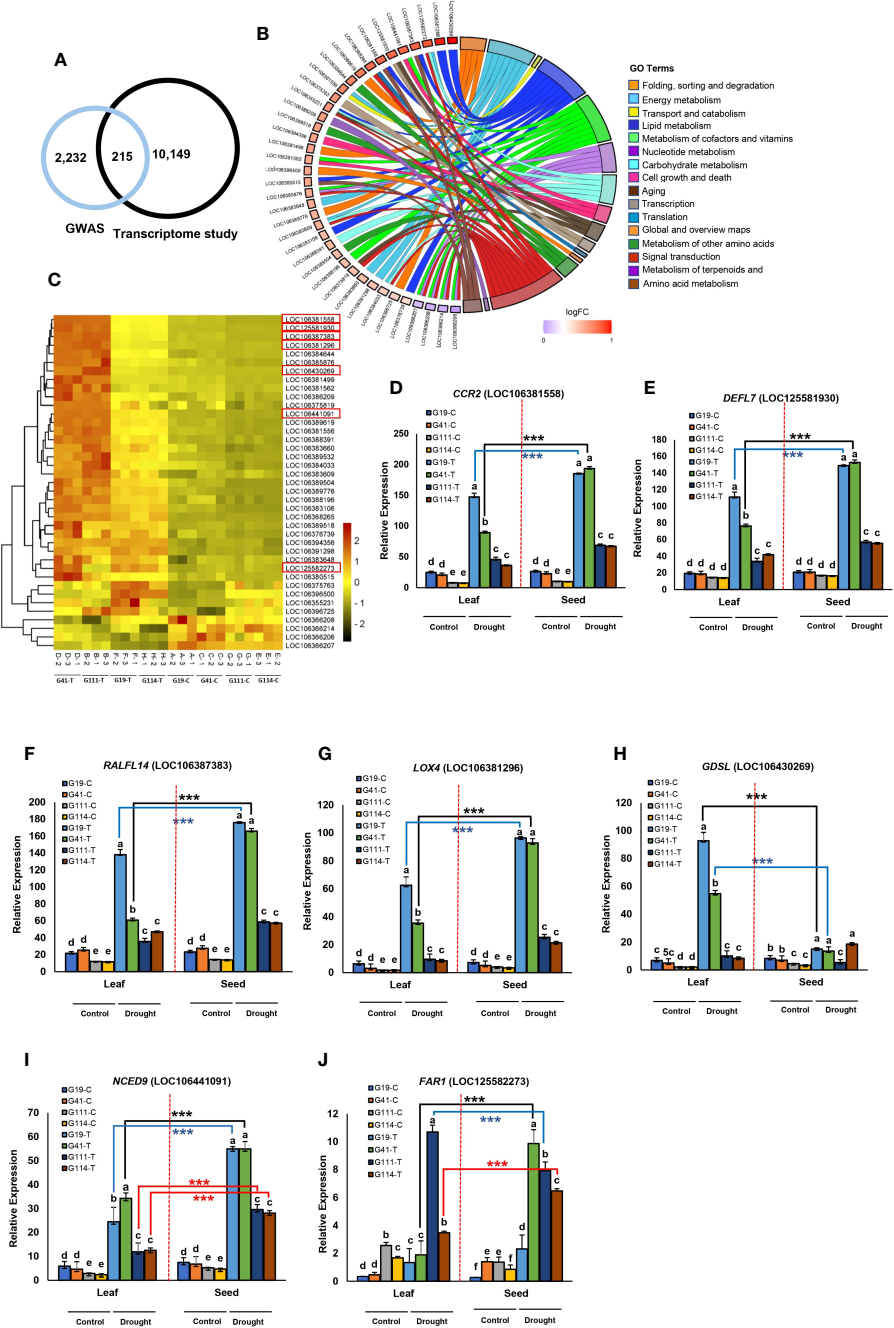
Analysis of transcriptomes between high- and low-yielding varieties selected in drought stress treatment in the field study. Transcriptome study for days to flowering (DTF). **(A)** Venn diagram of overlapped genes significant in GWAS and transcriptome analysis. **(B)** Detailed analysis of 16 enriched gene ontology groups selected using Circos plots for candidate genes in chromosomes A02, C01, C02, and C04 related to DTF. Symbols of DEG from each of the analyzed comparisons are displayed on the left side of the graph with their logFC values, mapped by color scale (red = higher expression; violet = lower expression). The white color corresponds to expression levels below the cutoff value for the given comparisons. Colored connecting lines determine gene involvement in the GO terms. **(C)** Heat map of the expression of 40 genes associated with DTF in chromosomes A02, C01, C02, and C04 among low-yield varieties (G111 and G114) and high-yield varieties (G19 and G41) under drought stress. The red box indicates the key genes in the associated region of C02 related to DTF. Heatmap color represents the expression level of each gene (rows) under drought treatments (fold change > 1, *P*-value < 0.05). Red bars: upregulation; green bars: downregulation; (C): control and (T): drought treatment. **(D-J)** Tissue-specific expression of seven candidate genes in the peak regions of C02 from four varieties of rapeseed (low-yield varieties: G111 and G114; high-yield varieties: G19 and G41) under drought stress; (C): control and (T): drought treatment. Tissue-specific expression of the gene *CCR2* **(D)**, *DEFL7* **(E)**, *RALFL14* **(F)**, *LOX4* **(G)**, *GDSL* **(H)**, *NCED9* **(I)**, and *FAR1* **(J)** between low-yield varieties and high-yield varieties under drought stress. Data are means ± SD, *P*-value < 0.05, as determined by multiple comparison testing by one-way ANOVA. Different letters indicate distinct groups. Red dotted line indicates division expression of genes between seed and leaf. Asterisks indicate significant difference between seed and leaf (Student’s *t*-test, ***P-value < 0.001).

Transcriptome sequencing was performed for further analysis of the identified CGs associated with the linked SNPs. We found that 215 CGs were significantly expressed under drought stress (**Additional File 11**; **Figure 6A**). The identified GCs were mainly associated with DTF (47.90% of the CGs), DTR (41.86%), PH (4.18%), and yield components (4.65%). The results of the KEGG pathway analysis are shown in **Figure 6B**. For the upregulated DEGs, 16 KEGG pathways were enriched according to *P*-value < 0.01 and FDR < 0.01. Our gene expression analysis identified 23 DTF-associated genes surrounding the SNP peaks in chromosome C02 of which 19 genes were upregulated under the DS condition (**Figure 6B**). One of these genes with 14.32-fold change between the two contrasting varieties, *GDSL ESTERASE/LIPASE* (LOC106430269) in C02, contained two significant DTF SNPs (**Figure 6C**). Six genes, *LOX4*, *FAR1*, *NCED9*, *CCR2*, *RALFL14*, and *DEFL7*, were located near our SNPs; Bn-scaff_18507_1-p354053, Bn-scaff_22749_1-p574003, Bn-scaff_16328_1-p636786, Bn-scaff_16485_1-p1575966, Bn-scaff_18245_1-p84866, and Bn-scaff_18406_1-p183669 were, respectively, upregulated in the DS condition and in the high-seed yield varieties (**Table 4**; **Figure 6C**). Among these genes, the *LOX4* gene showed 11.56-fold change between the two contrasting groups. In the KEGG analysis, we found that *GDSL ESTERASE/LIPASE* and *LOX4* are involved in the lipid metabolism pathways (**Additional File 12**; **Figure 6B**). Transcriptome analysis in both leaf and seed samples showed variable responses to drought stress. Higher DEGs and larger absolute changes were observed in expression of the genes in seed extracts than in leaf (**Figures 6D–J**).

**Table 4:** SNPs and candidate genes significantly associated with agronomic and yield-related traits integrating genome-wide association and transcriptome studies.

Traits	Lead SNP	Chr^a^	Position (bp)^b^	Allele	*P* value^c^	*R* ^2^ (%)^d^	Watering regime	Candidate Gene^e^	Annotation
DTF	Bn-scaff_18507_1-p354053	C02	23345227	A/G	8.15E-04	11.28	D17	LOC106381296	Lipoxygenase 4, chloroplastic (LOX4)
	Bn-scaff_18199_1-p285821	C02	24697490	A/G	8.15E-04	11.28	D17	LOC106430269	GDSL esterase/lipase At1g74460-like (GDSL)
	Bn-scaff_18199_1-p286255	C02	24697925	A/G	8.15E-04	11.28	D17		
	Bn-scaff_22749_1-p574003	C02	24986073	A/C	8.15E-04	11.28	D17	LOC125582273	Protein FAR1-RELATED SEQUENCE 3-like (FAR1)
	Bn-scaff_16328_1-p636786	C02	28382413	A/G	6.58E-04	11.62	D17	LOC106441091	9-cis-epoxycarotenoid dioxygenase NCED9, chloroplastic-like (NCED9)
	Bn-scaff_16485_1-p1575966	C02	29519291	A/G	6.58E-04	11.62	D17	LOC106381558	Cinnamoyl-CoA reductase 2-like (CCR2)
	Bn-scaff_18245_1-p84866	C02	30219143	A/G	6.58E-04	11.62	D17	LOC106387383	Protein RALF-like 14 (RALFL14)
	Bn-scaff_18406_1-p183669	C02	31546724	A/C	6.58E-04	11.62	D17	LOC125581930	Defensin-like protein 7 (DEFL7)
DTSD	Bn-A03-p27256355	A03	25485861	A/G	5.76E-04	16.57	D18	LOC111214287	Agamous-like MADS-box protein AGL19 (AGL19)
	Bn-A03-p27288521	A03	25524140	A/G	5.50E-04	16.67	D18		
	Bn-A03-p27289437	A03	25525060	A/C	7.95E-04	15.87	D18		
DTR	Bn-scaff_18936_1-p240670	C03	2852351	A/G	2.11E-05	24.55	W17	LOC125584437	Aspartic proteinase oryzasin-1-like (ORYZASIN1)
	Bn-scaff_18936_1-p269153	C03	2878346	A/G	1.36E-08	23.68	W17	LOC125584078	Aspartic proteinase oryzasin-1-like (ORYZASIN1)
	Bn-scaff_18936_1-p472353	C03	3058428	A/G	1.36E-08	42.99	W17	LOC106379137	Protein LAX PANICLE 2 (LAX2)
	Bn-scaff_18936_1-p559490	C03	3140112	A/G	1.07E-08	43.65	W17	LOC111204276	Flowering time control protein FY (FY)
	Bn-scaff_18936_1-p610540	C03	3182947	A/C	1.07E-08	43.65	W17	LOC106431084	Agamous-like MADS-box protein AGL15 (AGL15)
	Bn-scaff_18936_1-p610540	C03	3182947	A/C	1.07E-08	43.65	W17	LOC106431084	Agamous-like MADS-box protein AGL16 (AGL16)
PH	Bn-scaff_18936_1-p440619	C03	3023049	A/G	8.96E-06	27.75	W18	LOC106454811	Protein TIFY 9 (TIFY9)
	Bn-scaff_18936_1-p472353	C03	3058428	A/G	1.06E-05	27.34	W18	LOC106454850	4-substituted benzoates-glutamate ligase GH3.12 (GH3.12)
	Bn-scaff_18936_1-p559490	C03	3140112	A/G	1.06E-05	27.32	W18	LOC106431049	Polyamine oxidase 1 (PAO1)
	Bn-scaff_18936_1-p610540	C03	3182947	A/C	1.02E-05	27.42	W18		
	Bn-scaff_18936_1-p611810	C03	3184218	A/G	1.02E-05	27.42	W18		
BNPP	Bn-scaff_20901_1-p276384	C05	125848	A/G	4.90E-05	23.31	D17	LOC106358392	Subtilisin-like protease SBT1.1 (SBT1.1)
	Bn-scaff_21821_1-p122253	C05	135624	A/G	3.63E-05	20.96	D17		
	Bn-scaff_21821_1-p128045	C05	135780	A/G	3.63E-05	20.96	D17		
	Bn-scaff_20125_1-p116436	C05	136834	A/G	3.63E-05	20.96	D17		
	Bn-scaff_20125_1-p114833	C05	136975	A/G	3.63E-05	20.96	D17		
	Bn-scaff_20125_1-p110307	C05	11417447	A/G	1.31E-04	20.99	D17	LOC106347076	SKP1-like protein 3 (ASK3)
	Bn-scaff_20125_1-p110480	C05	11417644	A/G	3.63E-05	20.96	D17		
PW	Bn-scaff_19208_1-p94553	C04	35642269	A/G	6.23E-05	22.40	W20	LOC106391243	ABC transporter G family member 16-like (ABCG16)
	Bn-scaff_19208_1-p94501	C04	35642321	A/C	6.23E-05	22.40	W20		
	Bn-scaff_19208_1-p94498	C04	35642324	A/C	3.83E-05	23.55	W20		
	Bn-scaff_19208_1-p93814	C04	35643023	A/G	6.23E-05	22.40	W20		
SNPS	Bn-A02-p2124245	A02	751843	A/G	3.71E-04	18.61	D20	LOC106383096	MADS-box protein FLOWERING LOCUS C (FLC)
	Bn-A02-p2124328	A02	751923	A/G	5.18E-04	17.83	D20		
	Bn-A02-p2124421	A02	752016	A/G	2.88E-04	19.20	D20		
	Bn-A02-p2364479	A02	989812	A/C	2.66E-04	19.38	D20	LOC106383766	Protein BIG GRAIN 1-like D (BIG1-D)
	Bn-scaff_16069_1-p607537	C07	36944365	A/C	4.07E-04	15.68	D20	LOC106410807	Cullin-associated NEDD8-dissociated protein 1 (CAND1)
SWPP	Bn-A08-p13626189	A08	11377260	A/G	7.02E-04	17.23	D20	LOC125575103	Purine permease 21-like (PUP21)
	Bn-A08-p13626982	A08	11378053	A/G	7.76E-04	17.00	D20		
	Bn-A08-p13638847	A08	11390137	A/G	9.51E-04	16.53	D20	LOC106360860	Probable purine permease 10 (PUP10)
SY	Bn-scaff_19244_1-p283272	C01	224308	A/G	2.58E-05	24.345	D17	LOC106439041	Developmentally-regulated G-protein 3 (DRG3)
	Bn-scaff_19244_1-p517124	C01	449274	A/G	4.56E-04	17.693	D17	LOC125580497	Cytochrome P450 79B1 (CYP79B1)
	Bn-scaff_19244_1-p683537	C01	614927	A/G	4.68E-04	17.63	D17	LOC106426830	Agamous-like MADS-box protein AGL21 (AGL21)
	Bn-scaff_22148_1-p400066	C04	29470290	A/C	3.55E-05	23.587	D17	LOC106390665	Cytochrome P450 78A9 (CYP78A9)
	Bn-scaff_22148_1-p400140	C04	29470363	A/G	9.25E-06	23.587	D17		
	Bn-scaff_18776_1-p18005	C04	29479069	A/G	9.25E-06	23.587	D17		
	Bn-scaff_18776_1-p33555	C04	29490106	A/G	9.25E-06	23.587	D17		
	Bn-scaff_18776_1-p36364	C04	29492969	A/C	9.25E-06	23.587	D17		
	Bn-scaff_18776_1-p36455	C04	29493060	A/G	3.55E-05	23.587	D17		

DTF, DTR, PH, BNPP, PW, SWPP, and SY are the abbreviations of days to flowering, days to ripening, plant height, branch number/plant, plant weight, seed weight/plant, and seed yield, respectively. WW17, DS17, WW18, DS18, WW20, and DS20 are the codes of two watering regimes in 3 years: well-watered in 2017, drought stress in 2017, well-watered in 2018, drought stress in 2018, well-watered in 2020, and drought stress in 2020. ^a^Chromosome. ^b^Position in base pairs for the lead SNP according to version 4 of the rapeseed reference sequence. ^c^P-value of the corresponding agronomic and yield-related traits calculated by MLM (Mixed linear model). ^d^The phenotypic variance explained by the corresponding locus. ^e^A plausible candidate gene in the locus.

**Figure 7:**
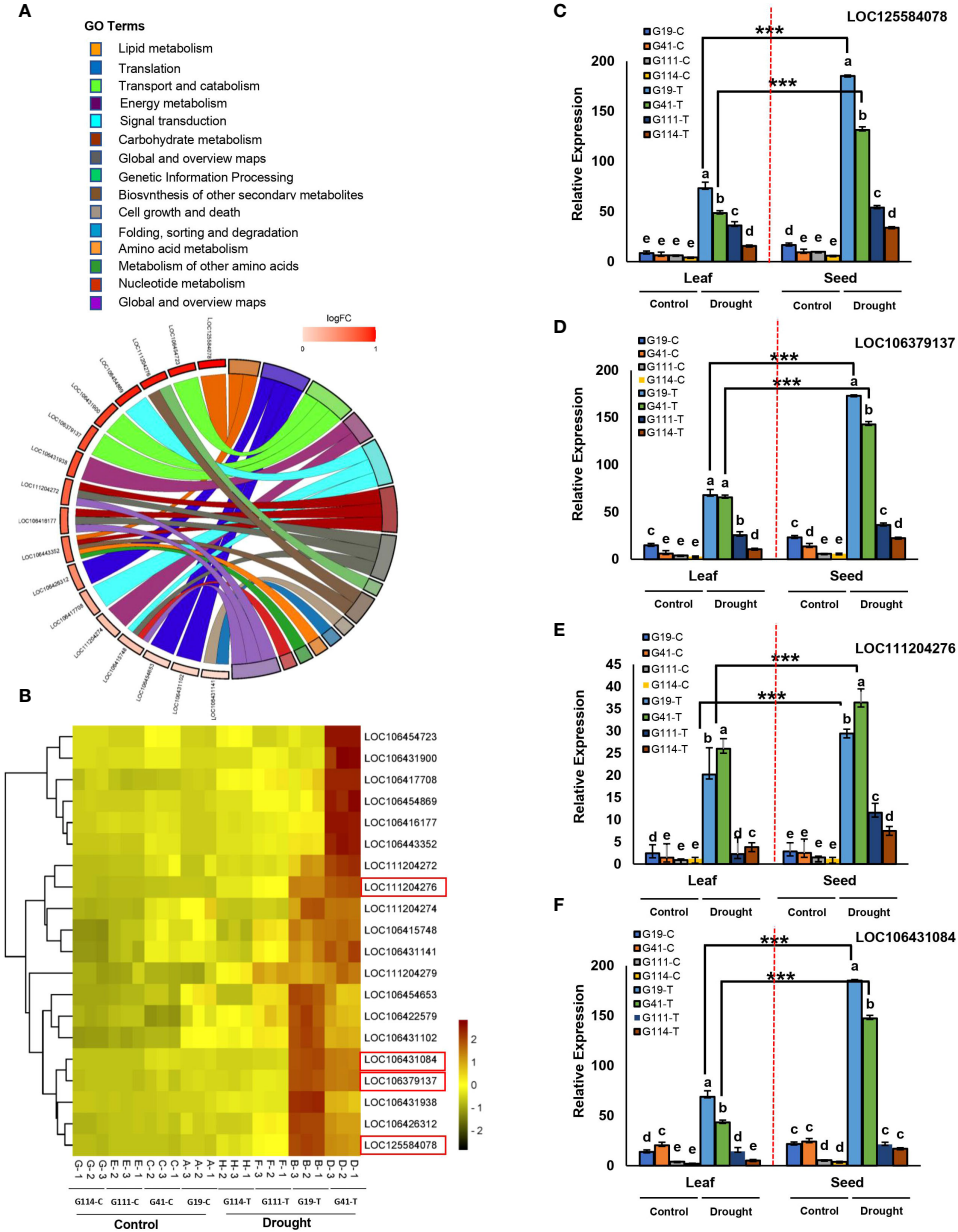
Analysis of transcriptomes between high- and low-yielding varieties selected in drought stress treatment in the field study. Transcriptome study for days to ripening (DTR). **(A)** Detailed analysis of 15 enriched gene ontology groups selected using Circos plots for candidate genes in chromosomes A01, C03, and C06 related to DTR. Symbols of DEG from each of the analyzed comparisons are displayed on the left side of the graph with their logFC values, mapped by color scale (dark red = higher expression; light red = lower expression). Colored connecting lines determine gene involvement in the GO terms. **(B)** Heat map of the expression of 20 genes associated with DTR in chromosomes A01, C03, and C06 among low-yield varieties (G111 and G114) and high-yield varieties (G19 and G41) under drought stress. The red box indicates the key genes in the associated region of C03 related to DTR. Heatmap color represents the expression level of each gene (rows) under drought treatments (fold change >1, *P*-value <0.05). Red bars: upregulation; green bars: downregulation; (C): control and (T): drought treatment. **(C-F)** Tissue-specific expression of seven candidate genes in the peak regions of C03 from four varieties of rapeseed (low-yield varieties: G111 and G114; high-yield varieties: G19 and G41) under drought stress; (C): control and (T): drought treatment. Tissue-specific expression of the gene LOC125584078 **(C)**, LOC106379137 **(D)**, LOC111204276 **(E)**, and LOC106431084 **(F)** between low-yield varieties and high-yield varieties under drought stress. Data are means ± SD, *P*-value < 0.05, as determined by multiple comparison testing by one-way ANOVA. Different letters indicate distinct groups. Red dotted line indicates division expression of genes between seed and leaf. Asterisks indicate significant difference between seed and leaf (Student’s *t*-test, *** *P*-value < 0.001).

We found that 20 genes surrounding the peak SNPs for DTR were significantly upregulated in the DS condition (**Figure 7B**). Expression of two genes, *ORYZASIN1* and *FY*, near the SNPs Bn-scaff_18936_1-p269153 and Bn-scaff_18936_1-p559490 for DTR, respectively, which was significantly higher in seed and drought than in leaf and WW conditions was significant between the two contrasting low- and high-yield varieties (**Figures 7B, C, E**). In the KEGG analysis, we found that *ORYZASIN1* and *FY* were from the lipid metabolism and biosynthesis of other secondary metabolites (**Figure 7A**; **Additional File 13**). The *LAX2* gene which belonged to transport and catabolism pathways was upregulated in drought treatment and contained a significant SNP in C03 for DTR (**Figures 7B, D**; **Table 4**; **Additional File 13**). Similar results were observed for gene expression level under drought stress for two members of the MADS domain family, *AGL15* and *AGL16*, that were near the significant SNP loci on C03 (**Table 4**). We observed that the expression of the *LAX2*, *AGL15*, and *AGL16* genes was significantly higher in seed than in leaf samples under drought (**Figure 7F**).

**Figure 8:**
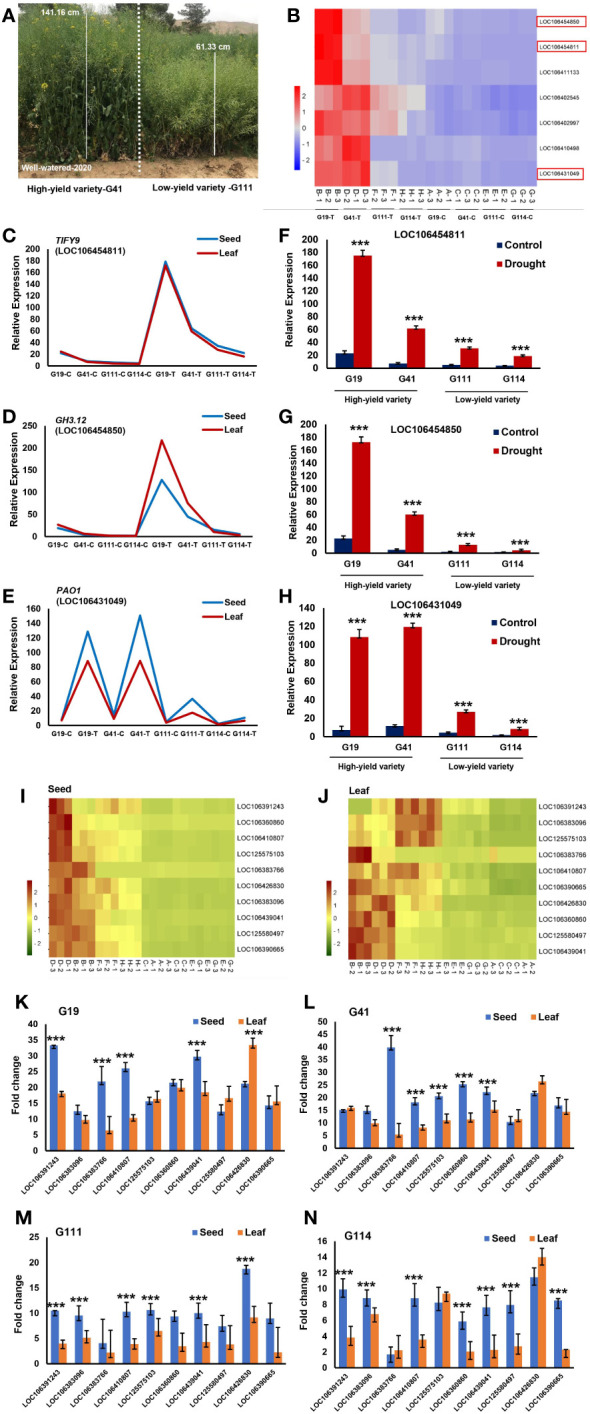
Transcriptome study for plant height (PH) and seed yield (SY). **(A)** Plant height phenotypes of two rapeseed varieties; G41 as a high-yield varieties and G111 as a low-yield varieties were grown in field under well-watered conditions in 2020. **(B)** Heat map of the expression of seven genes associated with PH in chromosomes A07, C03, C06, and C07 among low-yield varieties (G111 and G114) and high-yield varieties (G19 and G41) under drought stress. The red box indicates the key genes in the associated region of C03 related to PH. Heatmap color represents the expression level of each gene (rows) under drought treatments (fold change > 1, *P*-value < 0.05). Red bars: upregulation; blue bars: downregulation; (C): control and (T): drought treatment. **(C-E)** Tissue-specific expression of three candidate genes in the peak regions of C03 from four varieties of rapeseed (low-yield varieties: G111 and G114; high-yield varieties: G19 and G41) between seed and leaf under drought stress; (C): control and (T): drought treatment. Tissue-specific expression of the gene *TIFY9* **(C)**, *GH3.12* **(D)**, and *PAO1* **(E)** between seed and leaf from four varieties. **(F)** Expression pattern of the gene *TIFY9* between low-yield varieties and high-yield varieties under drought stress. **(G)** Expression pattern of the gene *GH3.12* between low-yield varieties and high-yield varieties under drought stress. **(H)** Expression pattern of the gene *PAO1* between low-yield varieties and high-yield varieties under drought stress. **(I, J)** Heat map of the expression of 10 key genes associated with SY in chromosomes A02, A08, C01, C04, and C07 among low-yield varieties (G111 and G114) and high-yield varieties (G19 and G41) from seed **(I)** and leaf **(J)** under drought stress. Heatmap color represents the expression level of each gene (rows) under drought treatments (fold change >1, *P*-value < 0.05). Red bars: upregulation; green bars: downregulation; (C): control and (T): drought treatment. **(K-N)** Tissue-specific expression of 10 genes associated with SY in four varieties: G19 **(K)**, G41 **(L)**, G111 **(M)**, and G114 **(N)** under drought stress. Asterisks indicate significant difference between seed and leaf (Student’s *t*-test, ***P-value < 0.001).

The *TIFY9*, *GH3.12*, and *PAO1* genes associated with PH SNPs on C03 showed significant differential expression between high- and low-yield varieties in the DS condition (**Figures 8A, B, E–H**). *GH3.12* had a higher expression in leaf than in seed (**Figure 8D**), whereas the expression of *TIFY9* was higher in seed (**Figure 8C**).

There were 10 DEGs (*BG1*, *FLC*, *DRG3*, *CAND1*, *PUP10*, *PUP21*, *ABCG16*, *AGL21*, *CYP79B1*, and *CYP78A9*) associated with the SY SNPs which showed significant differential expression between high- and low-yield varieties (**Table 4**; **Figures 8I–K**). These DEGs are known to regulate seed yield by affecting anther and pollen development, seed ripening, seed size, and seed weight regulation (Ma et al., 2015; Castelan-Munoz et al., 2019; Xu et al., 2019; Li et al., 2022). The *ABCG16* gene with 33.03-fold change in the seed sample showed a higher expression in the high-yield variety G19 under drought than in the WW condition (**Figures 8I–K**). The *AGL21* gene with 33.42-fold change showed higher expression in leaf under drought than in the WW condition in G19 (**Figure 8K**). The *CAND1* and *DRG3* genes had higher expression in seed than in leaf samples in both low- and high-yield varieties under drought conditions (**Figures 8I, K–N**).

### 3.10 Validation of major drought-related genes by combining GWAS and RNA-seq results

We identified the DEGs in the selected yield contrasting varieties in the drought compared with the well-watered treatment. A gene ontology enrichment analysis was also performed to identify the functional roles of DEGs and variety-specific responses under drought stress conditions. Transcriptomic analysis showed that 10 DEGs (*BG1*, *FLC*, *DRG3*, *CAND1*, *PUP10*, *PUP21*, *ABCG16*, *AGL21*, *CYP79B1*, and *CYP78A9*) had significantly higher expression in the high- than in low-yield varieties. These CGs were upregulated in the low-yield varieties under the drought stress compared with the well-watered conditions. Consistent with the higher number of DEGs observed in the leaf or seed of the high-yield varieties in response to drought, higher enriched GO terms unique to the high-yield varieties, particularly in seed, were detected. Most of the enriched GO terms exclusive to the high-yield varieties in seeds were those processes that were associated with abiotic stress, including the responses to ABA, osmotic adjustment, and regulation of stomatal movements. A number of 25 upregulated DEGs were identified in the seeds of the high-yield compared with 20 DEGs in the low-yield varieties (**Supplementary Figure S7**). In the high-yield varieties, xyloglucan endotransglucosylase/hydrolases (*XTH13*), triacylglycerol lipase (*SDP1*), serine/threonine-protein kinase (*SRK2F*), ABC transporter F family (*ABCF1*), ethylene-responsive transcription factor (*ERF113*), zinc-finger proteins (*ZC3H49*), F-box protein (*FBD*), NAC domain-containing proteins (*NAC072*), and acetyl-CoA acetyltransferase (*FadA*) showed above fivefold change (**Supplementary Figures S7C, E**). Upregulation of the *XTH13, NAC072* and *FadA* genes was observed in the two contrasting varieties (**Supplementary Figures S7C–F**). The *XTH* gene family is involved in various physiological processes in plants, especially in abiotic stress responses and cell elongation (Ma et al., 2022). The *XTH* genes have been regulated by TF NAC by directly binding to the promoter region of the *XTH* genes improving stress tolerance (Tao et al., 2022).

## 4 Discussion

### 4.1 Traits variations in rapeseed under drought stress

Breeding rapeseed which is an important source of oil and protein for food and industrial applications is challenging due to environmental stresses worldwide (Elferjani and Soolanayakanahally, 2018; Secchi et al., 2023). Results of evaluation of a rapeseed population over three growing seasons showed large genetic diversity for morphological, phenological, and yield traits in both drought and well-watered treatments. Our data showed a significant treatment by trait value interaction. Phenotyping under two irrigation regimes revealed differences for the traits among varieties over treatments and years that were higher than those in earlier studies (Korber et al., 2016; Luo et al., 2017; Raman et al., 2020; Menendez et al., 2021; Qin et al., 2022; Yang et al., 2023). Higher heritability and genetic gain from selection for several traits in this study under two contrasting irrigation regimes indicated selection potential for the improvement of drought tolerance in the tested rapeseed population. Our study showed that the 119 varieties could be clustered into seven supported genetic lineages that roughly reflected their geographical origin consistent with previous studies (Gazave et al., 2016; Bird et al., 2017).

### 4.2 Novel and stable loci identified for agronomic and yield-related traits under drought conditions

Due to the sensitivity of complex traits to the environmental effects, the integration of GWAS and RNA-seq across different tissues might assist identification of candidate genes responsible for stress tolerance. Our study uncovered numerous loci correlated with variation in agronomic and yield-related traits. This study identified a set of candidate genes that could be exploited to alter agronomic traits and yield components to improve grain yield in rapeseed varieties. Life cycle timing is critical for yield and productivity of *Brassica napus* cultivars grown in different environments. Timing of flowering is crucial for optimal pollination, survival in specific environments, high seed quality, and seed yield and maintaining seed propagation in crop rotation systems (Kirkegaard et al., 2018). In the present study, we identified six stable SNPs for flowering time that were common between the two irrigation treatments across years. In addition, 171 novel signals significantly associated with flowering time were identified under drought conditions. Numerous loci of flowering time in *B. napus* have been identified on chromosomes A02, C02, and C03 by QTL mapping and GWAS in other studies (Akhatar et al., 2021; Vollrath et al., 2021; Han et al., 2022). We identified 21 novel MTAs on chromosome C03 for ripening time in rapeseed that were not previously reported. In temperate regions such as Iran, early flowering and maturity are important breeding targets in *B*. *napus* because drought restricts the growth season (Helal et al., 2021). Identification of candidate loci involved in drought tolerance can be used for marker-assisted selection and helps develop drought-tolerant rapeseed for dry regions. In a meta-QTL analysis, co-localized QTLs for flowering and maturity times were identified on chromosomes A01, A02, A03, A05, and C09 (Zhou et al., 2014).

Plant architecture (PA), which refers to the spatial distribution pattern of aboveground parts including plant height (PH) and number of aerial branches, is influenced by both genetic and environmental factors (Cai et al., 2016; Wang et al., 2019; Dong et al., 2022). In the present study, we identified 70 unique SNPs on chromosomes A01, A02, A04, A07, A09, A10, C03, and C05 associated with PH under two irrigation regimes of which several SNPs were close to those identified in previous studies (Sun et al., 2016a; Zhang et al., 2023a). In line with results of the Liu et al. (2021) and Zheng et al. (2017) studies, the highest number of significant SNPs for PH was identified on chromosome C03. In the current study, we identified 22 unique SNPs associated with branch number under drought stress condition that were not discovered in previous studies in rapeseed. These SNPs provide new information for understanding the establishment of ideal PA and developing breeding strategies for yield improvement in rapeseed. The molecular genetic mechanisms underlying branch number have been analyzed using GWAS in other rapeseed studies (Sun et al., 2016b; Dong et al., 2022; Hu et al., 2022). In the Sun et al. (2016b) study, 56 unique loci significantly associated with branch number explained up to 51.1% of the phenotypic variation. Hu et al. (2022) identified two significant SNP signals on chromosome C07 associated with branch trait in three environments.

Increasing the yield potential is a major goal for rapeseed breeding. We identified SNPs linked with yield and yield components of which several were stable over years and unique to this study. We identified 10 significant association signals for each THSW and SNPS traits that were stable across 2 of 3 years under drought stress conditions. Among the linked SNPs, 37 associated with two or more traits. Our pleiotropic SNP Bn-A02-p3539297 affecting both DTF and DTSD traits in our study has also been detected in a GWAS in the Xu et al. (2016) study. The position of the pleiotropic SNP Bn-A10-p13390065 affected DTF and DTSD in our study was close to the position of four flowering time-related genes (*BnaA10g18420D*, *BnaA10g18480D*, *BnaA10g22080D*, and *BnaA10g24300D*) identified in the Helal et al. (2021) study. Markers stable over environments and those with pleiotropic effects are preferred for use in marker-assisted selection (MAS) programs. According to the SNP-trait associations, we identified 188 SNPs for SY of which the SNPs in the 34,329-kbps–34,381-kbps interval on chromosome C06 were adjacent to the position of previously reported SNPs associated with yield components (Pal et al., 2021). In addition, the SNP Bn-A08-p14538807 on chromosome A08 was close to the SNP Chr18:12100271 detected for siliqua length in Pal et al. (2021). The SNPs in the 131-kbps–677-kbps interval on chromosome C01 overlapped with the position of the linked SNPs detected by Zhang et al. (2011) and Dong et al. (2018). In this study, 38 SNPs for HI were identified under two irrigation regimes across 3 years of which the SNPs Bn-scaff_16361_1-p2541607 and Bn-scaff_16361_1-p2545776 on chromosome C08 were close to the SNP Chr18:33618188 detected for HI in Qin et al. (2022).

### 4.3 Candidate genes involved in rapeseed growth under drought stress

Information about genetic control of architecture- and phenology-related characters helps in breeding for drought tolerance and avoidance. Integration of GWAS and RNA-seq revealed 59 DEGs associated with flowering and maturity times of which the *LOX4, GDSL*, *FAR1, NCED9, CCR2, RALFL14, DEFL7*, *ORYZASIN1*, *LAX2*, *FY*, *AGL15*, *AGL16*, and *AGL19* genes were key flowering and maturity genes in our study. In the hormone pathway, the *LOX4*, *NCED9*, *ORYZASIN1*, and *LAX2* genes play important roles in flower development and pollen tube growth (Caldelari et al., 2011; Chauvin et al., 2013; Huang and Han, 2014; Gomez et al., 2015; Li et al., 2018; Zuniga-Mayo et al., 2018; Park et al., 2019; Tran et al., 2023; Zhang et al., 2023d). From the auxin carrier AUX/LAX family, *LAX2* regulates the floral organ development by modulating auxin polar transport (Cardarelli and Cecchetti, 2014). Auxin, which is required for floral meristem initiation, regulates floral organ initiation, growth, and patterning that ensure reproductive success of the mature flower (Iqbal et al., 2017). In the present study, we also identified three members of MADS-box genes, *AGL15*, *AGL16*, and *AGL19*, that were involved in flowering and maturity in rapeseed. *FAR1, CCR2*, *AGL15*, *AGL16*, and *AGL19* regulate several events during pollen development such as tapetal degradation, the formation of anther cuticle and pollen exine, central vacuole development, and flowering transition (Fang and Fernandez, 2002; Lee and Lee, 2010; Whittaker and Dean, 2017; Ma et al., 2020; Zhao et al., 2020; Li et al., 2021; Chen et al., 2022). It has been shown that *FAR1* involved in floral bud differentiation interacts with proteins of flowering promoting SQUAMOSA-PROMOTER BINDING PROTEIN-LIKE (SPL) transcription factors (Xie et al., 2020). *FAR1* is even involved in branching regulation and floral bud sex differentiation (Hiraoka et al., 2013). Plant GDSL lipases form a large gene family, and members have been identified in *Arabidopsis* (105), *Oryza sativa* (114), and *Brassica rapa* L. (121) (Lai et al., 2017; Shen et al., 2022), which regulate seed development (Ma et al., 2018; Ding et al., 2019; Zhang et al., 2020; Liu et al., 2023).

Seven DEGs including *TIFY9*, *GH3.12*, and *PAO1* were identified as the candidate genes of plant height in the present study. *TIFY* genes play an important role in leaf and stem growth and responses to environmental stresses (Vanholme et al., 2007; Cai et al., 2014; Baekelandt et al., 2018; Liu et al., 2020a; Singh and Mukhopadhyay, 2021). The overexpression of *TIFY1* in *Arabidopsis* resulted in elongated petioles and hypocotyls due to increased cell elongation (Shikata et al., 2004). Previous studies have shown that the *BrGH3.12*, one of the CGs identified in our study, was highly expressed in the leaf apical region, which regulate flowering in *B*. *rapa* (Gu et al., 2017). It has been shown that upregulation of *BnaC03.GH3-12* may improve stress tolerance ability in *B*. *napus* (Wang et al., 2019). Polyamine oxidase (PAO) has been detected in many actively growing tissues (roots, stems, leaves, and floral organs) and plays a key role in maintaining normal growth and resisting the adverse environmental stresses in plants (Murray Stewart et al., 2018; Yu et al., 2019; Wu et al., 2022b; Samanta et al., 2023).

### 4.4 Genes responsible for yield variation between the high- and low-yield varieties under drought conditions

The number of seeds per silique and seed size/seed weight traits are the important goals of *B*. *napus* breeding and development of new high-density seeding varieties (Zhu et al., 2023). In the present study, 10 DEGs were identified as the yield component-related CGs of which *BIG GRAIN 1* (*BG1*) governs seed size in legume species (Ge et al., 2016) and rice (Lo et al., 2020). The expression level of *BG1* and *PUP10* in the high-yield varieties was higher than in low-yield varieties, which suggests that *BG1* and *PUP10* might positively regulate the grain yield. The *BG3* gene encoding a purine permease regulates grain size *via* modulating cytokinin (CK) transport in rice (Xiao et al., 2019). Among the CGs detected in our study, long- and short-distance transporters including *ABCG16*, *PUP10*, and *PUP21* are from the ABC transporter and PUP families that are involved in CK traffic (Gillissen et al., 2000; Tsai et al., 2012; Xiao et al., 2019; Zhao et al., 2019; Liu et al., 2020b).

We identified *CYP78A9* and *CYP79B1* as two members of the CYP family genes that play highly conserved roles in facilitating organ growth including floral organ, seeds, embryos, and endosperm in *Arabidopsis*, maize, soybean, and rice (Sotelo-Silveira et al., 2013; Wang et al., 2015b; Xu et al., 2015; Sun et al., 2017; Yeon et al., 2021). Shi et al. (2019) reported that the expression level of *BnaA9.CYP78A9* in silique valves of the long-silique variety was much higher than that in the regular-silique variety and that the long-silique plants showed higher concentrations of auxin in the developing silique, suggesting that *BnaA9.CYP78A9* contributes to the silique elongation phase in rapeseed.

The expression level of *cullin-associated NEDD8-dissociated protein 1* (*CAND1*) and *developmentally regulated G-protein 3* (*DRG3*) genes associated with yield components in our study was much higher in seed than in leaf of high-yield varieties. One of the critical elements of plant reproduction is the production of functional pollen grains. The expression of *CAND1* which is one of the key regulators of the SKP1-CUL1/RBX1-F-BOX (SCF) complex is imperative for fertility (Feng et al., 2004; Li et al., 2022; Rani et al., 2023). *CAND1* regulates the dynamic and functionality of the SCF complex, which is required for pollen and grain development) Hong et al., 2014(. Analysis of the *cand1-3* mutants in the Li et al. (2022) study has shown that *CUL1* from the SCF complex was expressed in pollen at a level significantly higher than any other tissue in *Arabidopsis* that could be due to the high concentration of the SCF substrates in pollen. *DRG3*, one of the CGs in our study, is a member of the family of GTP binding protein (G-proteins). G-proteins regulate plant growth and development pathways especially phytohormone signaling and cross-talk and defense responses (Kumar et al., 2014; Pandey, 2020). G-proteins are also known to regulate key agronomical traits such as seed size and yield (Cui et al., 2020). Roy Choudhury et al. (2014) identified the correlation between plant-specific G protein expression in seed tissue with higher seed size, seed mass, and seed number per plant, effectively resulting in significantly higher seed yield in *Camelina sativa*.

### 4.5 Candidate genes associated with enhanced drought tolerance in rapeseed

Breeding drought-tolerant rapeseed has always been a tough challenge for plant breeders. The use of grain yield as a selection criterion has proved to be a boon in this area of research. In addition, understanding response to drought stress in high-yielding crops at the molecular level is useful for developing drought tolerance. In this study, we found the *XTH13*, *SDP1*, *SRK2F*, *ABCF1*, *ERF113* genes that were upregulated in the high-yield varieties under drought conditions. Xyloglucan endotransglucosylase/hydrolase (XTH) is one of the critical enzymes which contribute to the development and strengthening of cell walls (Shi et al., 2015). The constitutive expression of *XTH* that increased stomatal closures was conferred by the increased cell-wall remodeling activity of *XTH* in guard cells, which may reduce transpirational water loss in response to dehydration stress (Choi et al., 2011; Han et al., 2017). In plants, the SnRK family comprises 38 members, which can be subdivided into three subfamilies: SnRK1, SnRK2, and SnRK3. Compelling evidence suggests that SnRK2s are involved in ABA and/or stress signaling pathways. The SnRK2 protein kinase is a central regulator of abscisic acid (ABA)-dependent stomatal closure (Kamiyama et al., 2021; Hasan et al., 2022). Some SnRK2 are also activated by hyperosmotic stress (Julkowska et al., 2015; Hasan et al., 2022). *ERF113* is a member of the ethylene response factor (ERF) family that is induced by salt stress and drought stresses (Debbarma et al., 2019). Additionally, *ERF113* transcription is responsive to jasmonic acid, salicylic acid, ABA, and ethylene hormones for stress tolerance (Krishnaswamy et al., 2011). In the Fang et al. (2022) study, transgenic soybean plants overexpressing *ERF113* showed significantly slower water loss in the leaves than wild type and plants with RNAi silencing of the gene under drought stress. These results reveal that the *XTH13*, *SDP1*, *SRK2F*, *ABCF1*, and *ERF113* genes identified in our study might improve drought tolerance in rapeseed, providing a theoretical basis for the molecular breeding of drought-tolerant varieties.

## 5 Conclusion

In the present study, multiomics analysis with the use of phenotypic, genomic, and transcriptomic data was performed to identify the major QTNs/genes associated with the plant responses to the effects of drought stress in rapeseed. We found high variation and linked SNPs for agronomic and seed yield-related traits that can be used in marker-assisted breeding (MAB) and development of improved rapeseed varieties with high yield under drought stress conditions. Two SNPs (Bn-scaff_18936_1-p610540 and Bn-scaff_18936_1-p472353) on chromosome C03 explained 43.65% and 42.99% of the phenotypic variance of ripening time, which is an important drought-adaptive trait. There were 10 drought tolerance-related genomic regions located on chromosome A02 (750,194 bp–791,056 bp) that had not been reported in previous studies in rapeseed. Four novel SNPs on chromosome C04 for plant weight were located within the sequence of the drought tolerance-related gene *ABCG16*, and their pleiotropic effects on seed weight per plant and seed yield were characterized. The GWAS analysis showed that 49 pleiotropic SNPs on chromosomes A01, A02, A08, A10, C03, and C04 were associated with at least two traits and 10 SNPs had a stable association with thousand seed weight overs 2 years under drought stress conditions. Overall, our study provides supporting information for three research areas. One is an irrigation treatment by trait value interaction and the interrelationship of novel candidate genes and drought-adaptive traits that help to unravel the drought tolerance mechanisms in rapeseed. The other is comparative transcriptomics that proved that seed weight/plant and plant weight at the phenotypic level and stress signaling pathway genes at the molecular level had higher contributions in response to the high-yield varieties to drought stress. The third is a large SNP data set correlated with drought tolerance, which will accelerate future efforts aiming at the development of drought-tolerant varieties through a MAS program.

## Data availability statement

The datasets presented in this study can be found in online repositories. The names of the repository/repositories and accession number(s) can be found in the article/**Supplementary Material**.

## Author contributions

MS: Investigation, Writing – original draft, Data curation, Formal analysis, Methodology, Software. BH: Investigation, Writing – original draft, Conceptualization, Project administration, Supervision, Visualization, Writing – review & editing. BA: Investigation, Methodology, Writing – review & editing. JB: Data curation, Investigation, Methodology, Writing – review & editing. JW: Data curation, Investigation, Visualization, Writing – review & editing. XT: Data curation, Investigation, Visualization, Writing – review & editing. AD: Writing – review & editing. CR: Visualization, Writing – review & editing.
