# Supplementary material for: Dissection of quantitative trait nucleotides and candidate genes associated with agronomic and yield-related traits under drought stress in rapeseed varieties: integration of genome-wide association study and transcriptomic analysis
Source: Front Plant Sci. 2024 Mar 19;15:1342359. doi: 10.3389/fpls.2024.1342359 (PMC10985355; doi:10.3389/fpls.2024.1342359)
Supplement: Supplementary file 1 [file DataSheet_1.pdf]

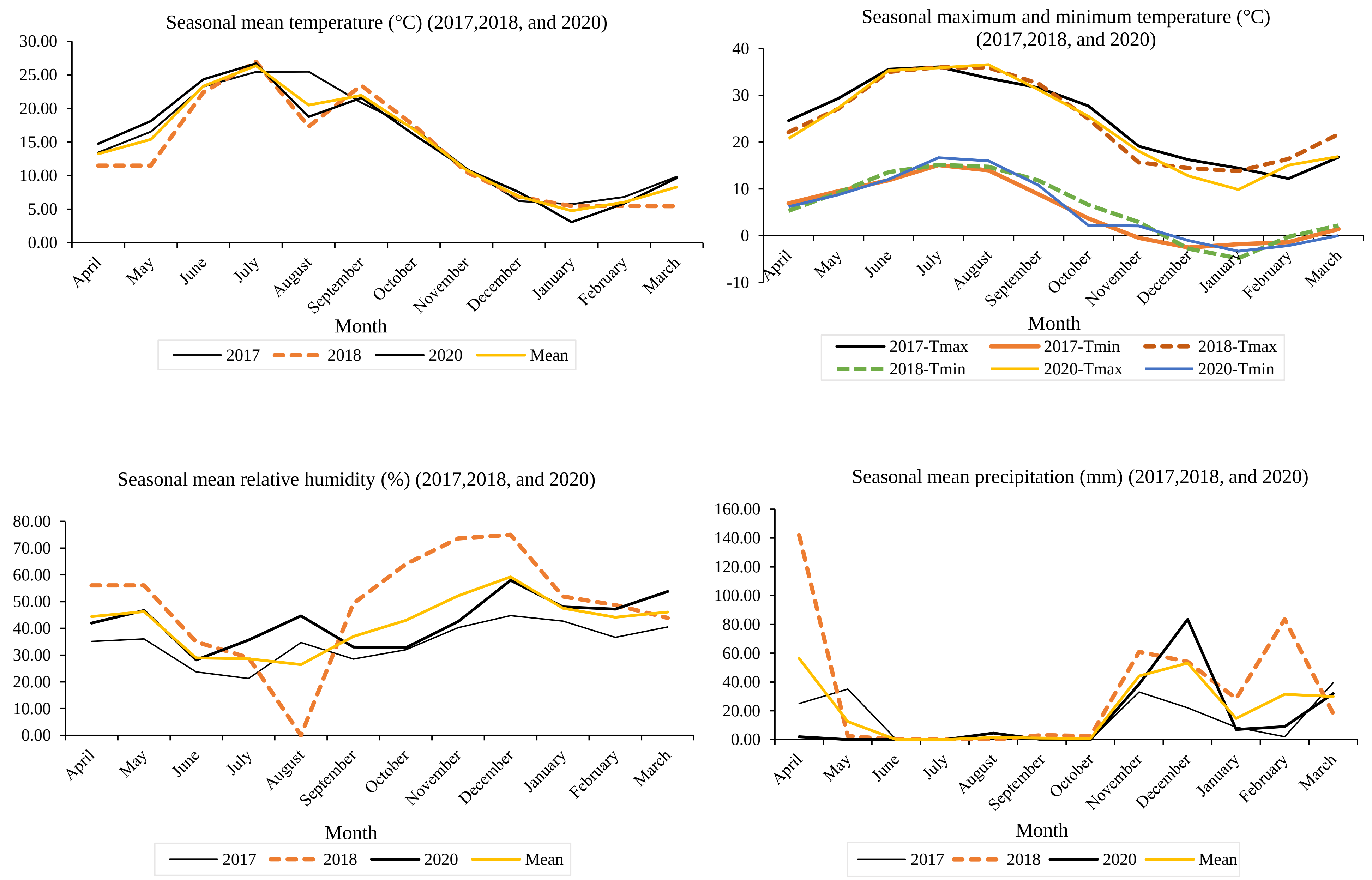

Supplementary Figure S1 Seasonal minimum, maximum and mean temperature, seasonal mean values of air relative humidity, and seasonal mean precipitation during the rapeseed (*Brassica napus*) growing season across three years (2017, 2018, and 2020).

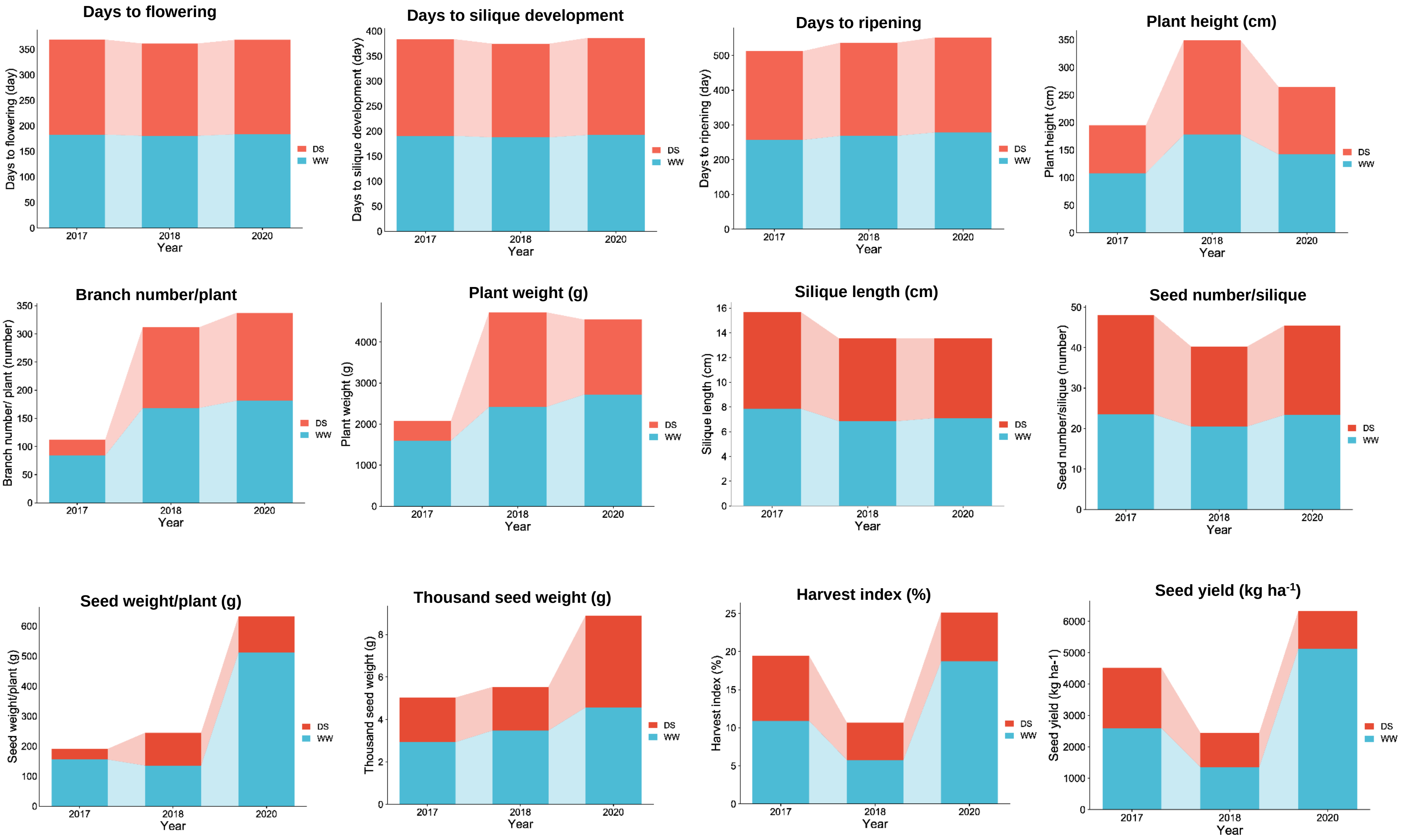

Supplementary Figure S2 Effects of drought stress on agronomic and yield related traits in rapeseed (*Brassica napus*) across three years (2017, 2018, and 2020). Vertical axes represent proportions. Blue and red column represent well-watered and drought stress conditions, respectively.

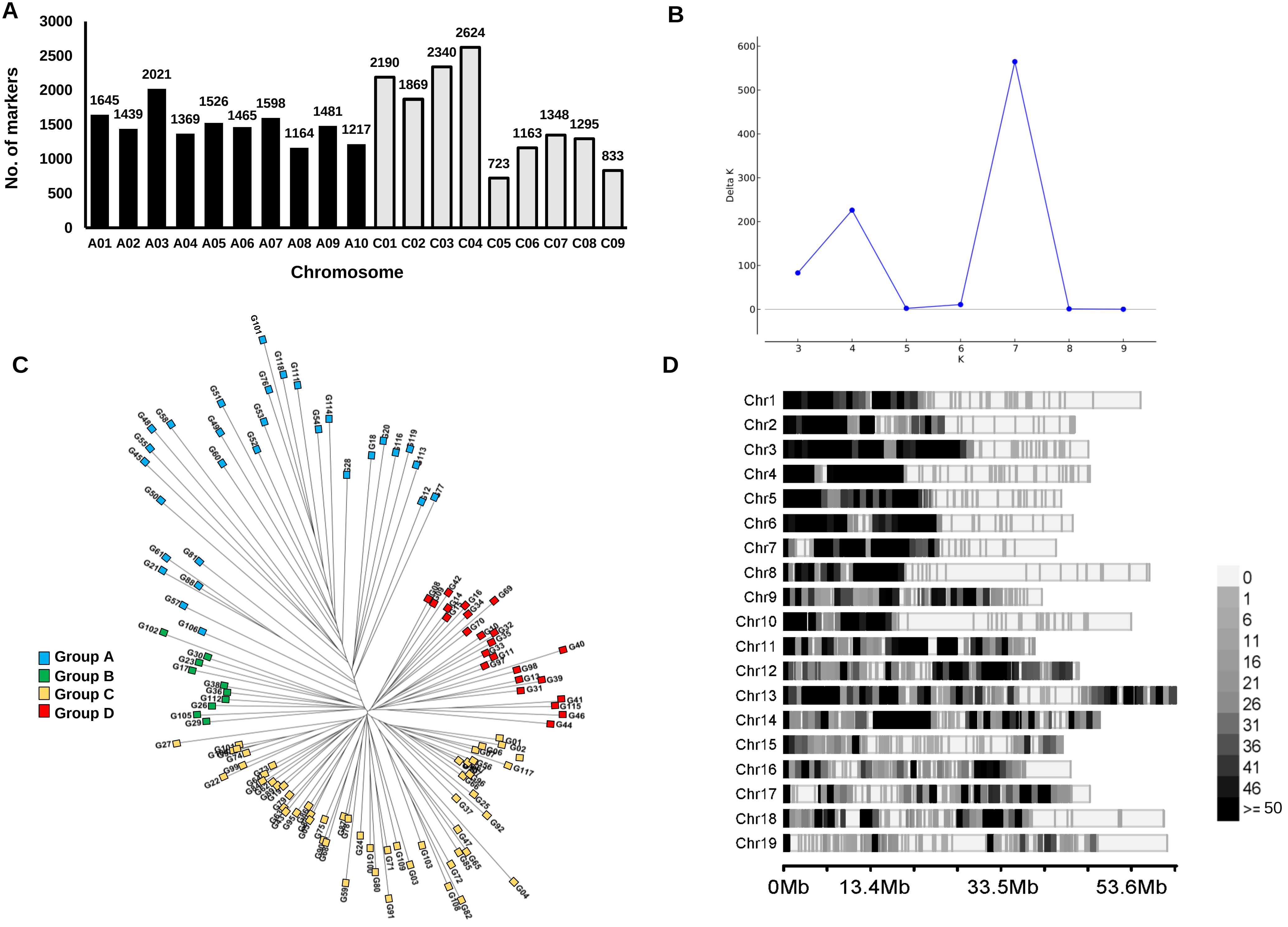

A
No. of markers
3000
2500
2000
1500
1000
500
0
1645
1439
2021
1369
1526
1465
1598
1164
1481
1217
2190
1869
2340
2624
723
1163
1348
1295
833
A01 A02 A03 A04 A05 A06 A07 A08 A09 A10 C01 C02 C03 C04 C05 C06 C07 C08 C09
Chromosome
B
Delta K
600
500
400
300
200
100
0
3 4 5 6 7 8 9
K
C
Group A
Group B
Group C
Group D
D
Chr1
Chr2
Chr3
Chr4
Chr5
Chr6
Chr7
Chr8
Chr9
Chr10
Chr11
Chr12
Chr13
Chr14
Chr15
Chr16
Chr17
Chr18
Chr19
0Mb
13.4Mb
33.5Mb
53.6Mb
0
1
6
11
16
21
26
31
36
41
46
>= 50

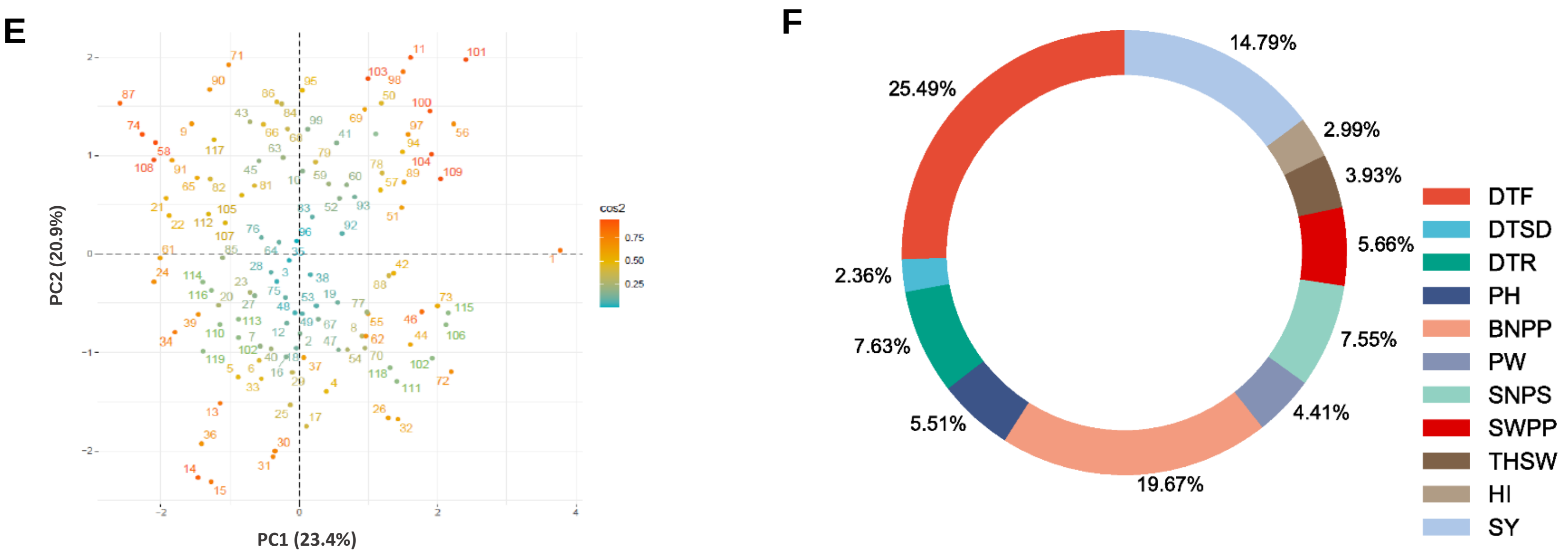

Supplementary Figure S3 (A) Distribution of 29,310 SNP markers across different chromosomes of rapeseed (*Brassica napus* L.). (B) Delta K values for different numbers of populations assumed (K) in the STRUCTURE analysis. (C) Neighbor-joining (NJ) cluster analysis of 119 rapeseed varieties inferred from 29,310 SNPs. G01-G119 stand for code of the rapeseed varieties. (D) Heatmap for distribution of 29,310 SNPs within 1 Mb window size in the rapeseed. The horizontal axis shows the chromosome length (Mb). (E) PCA plots of first two components of 29,310 SNPs in 119 rapeseed varieties. (F) *B. napus* single nucleotide polymorphism (SNP)-trait association chart under two watering regimes (well-watered and drought stress conditions) across three years (2017, 2018, and 2020). DTF, DTSD, DTR, PH, BNPP, PW, SNPS, SWPP, THSW, HI, and SY were the abbreviations of days to flowering, days to silique development, days to ripening, plant height, branch number/plant, plant weight, seed number/silique, seed weight/plant, thousand seed weight, harvest index, and seed yield.

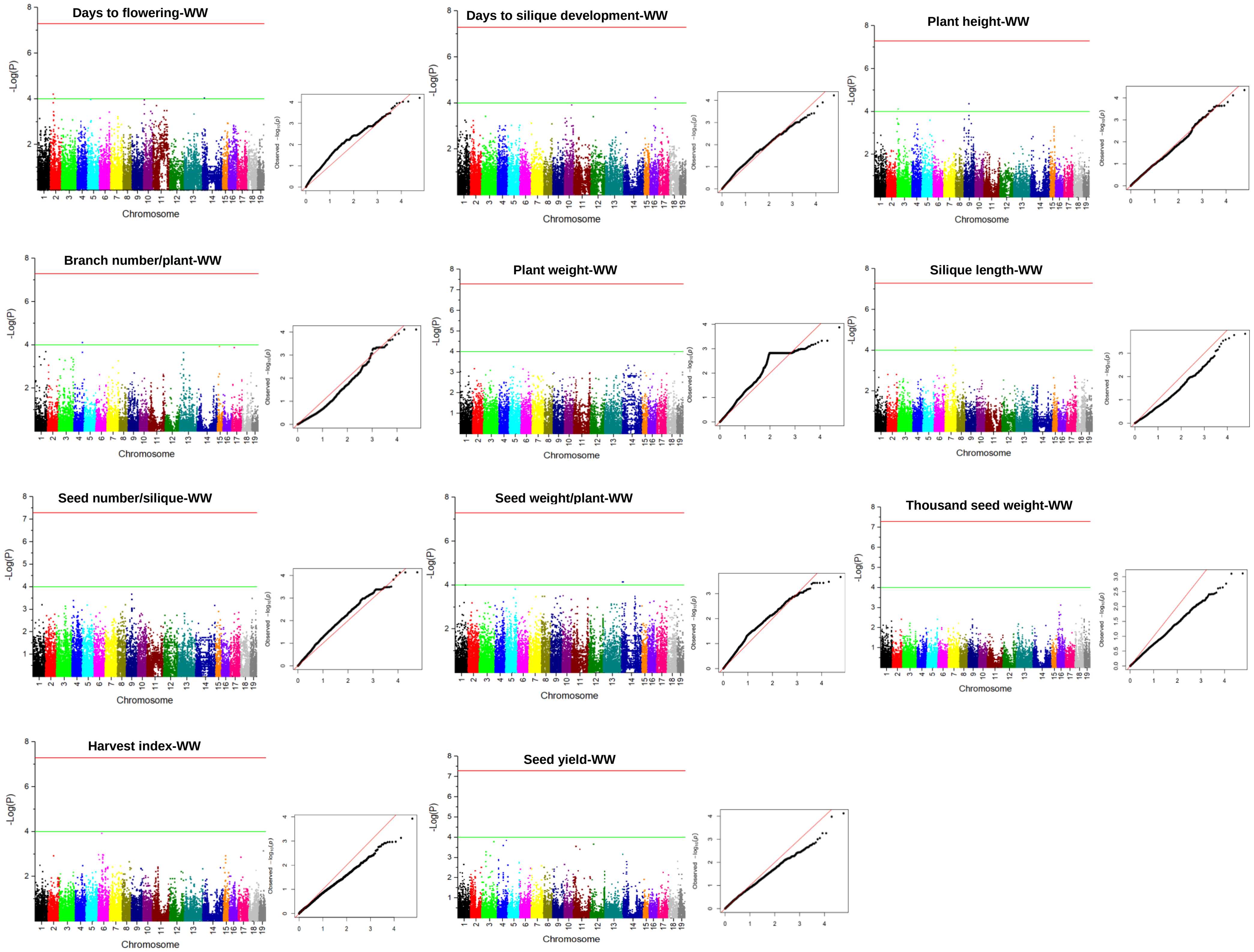
Days to flowering-WW
Days to silique development-WW
Plant height-WW
Branch number/plant-WW
Plant weight-WW
Silique length-WW
Seed number/silique-WW
Seed weight/plant-WW
Thousand seed weight-WW
Harvest index-WW
Seed yield-WW
-Log(P)
Chromosome
Observed -log10(p)

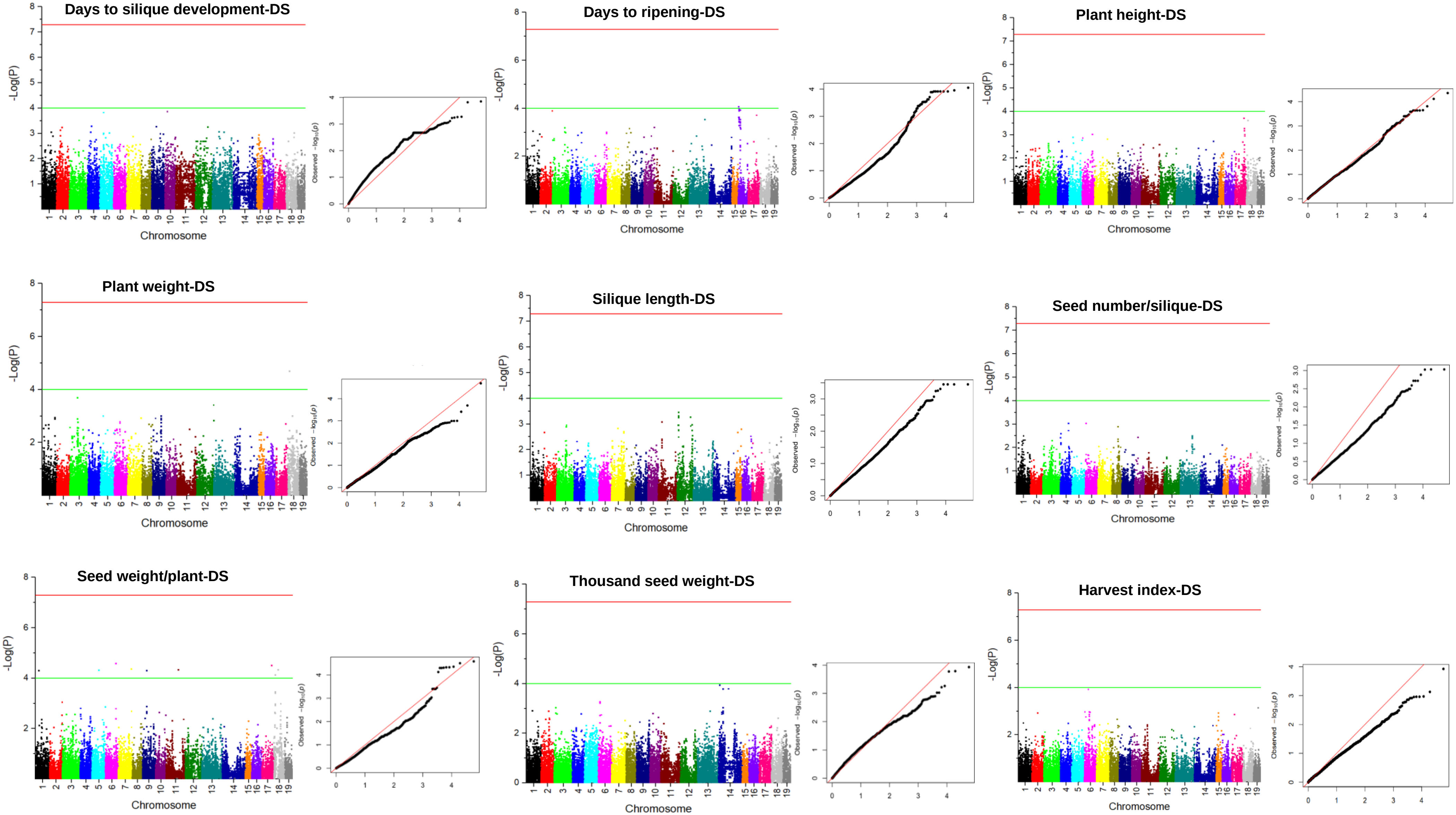
Days to silique development-DS
Days to ripening-DS
Plant height-DS
Plant weight-DS
Silique length-DS
Seed number/silique-DS
Seed weight/plant-DS
Thousand seed weight-DS
Harvest index-DS
-Log(P)
Chromosome
Observed $-\log_{10}(p)$

Supplementary Figure S4 Genome-wide association-mapping results for agronomic and yield related traits in rapeseed (*Brassica napus*) using single-trait analysis in all accessions grown in season 2017. Manhattan plot and corresponding quantile-quantile (QQ) plot displaying the GWAS result of agronomic and yield related traits in 19 chromosomes at well-watered (WW) and drought stress (DS) conditions. In the QQ plot, the x-axis displayed the expected –log10 *p*-value and y-axis represented the observed –log10 *p*-value.

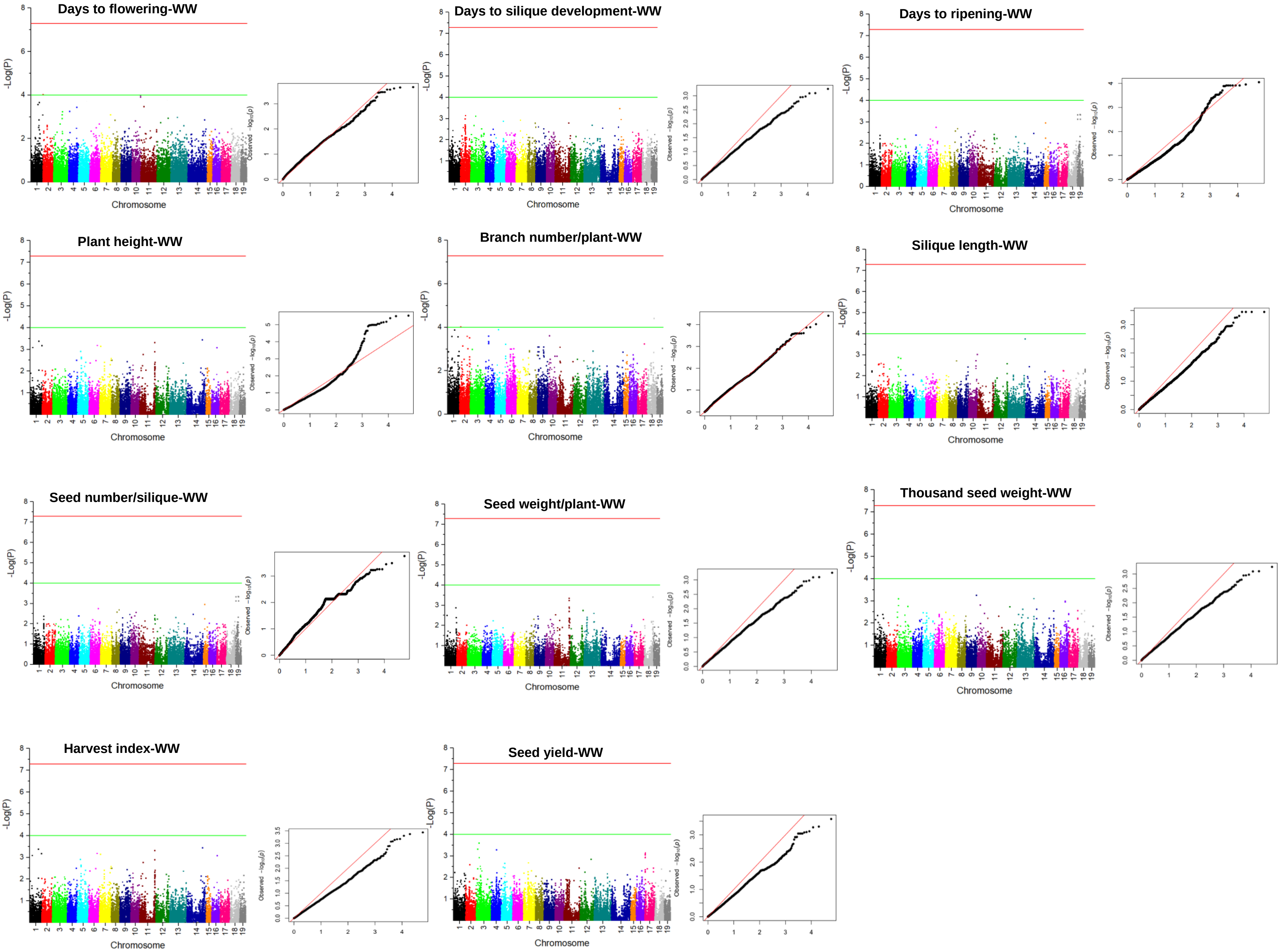

Days to flowering-WW
Days to silique development-WW
Days to ripening-WW
Plant height-WW
Branch number/plant-WW
Silique length-WW
Seed number/silique-WW
Seed weight/plant-WW
Thousand seed weight-WW
Harvest index-WW
Seed yield-WW
-Log(P)
Chromosome
Observed −log10(p)

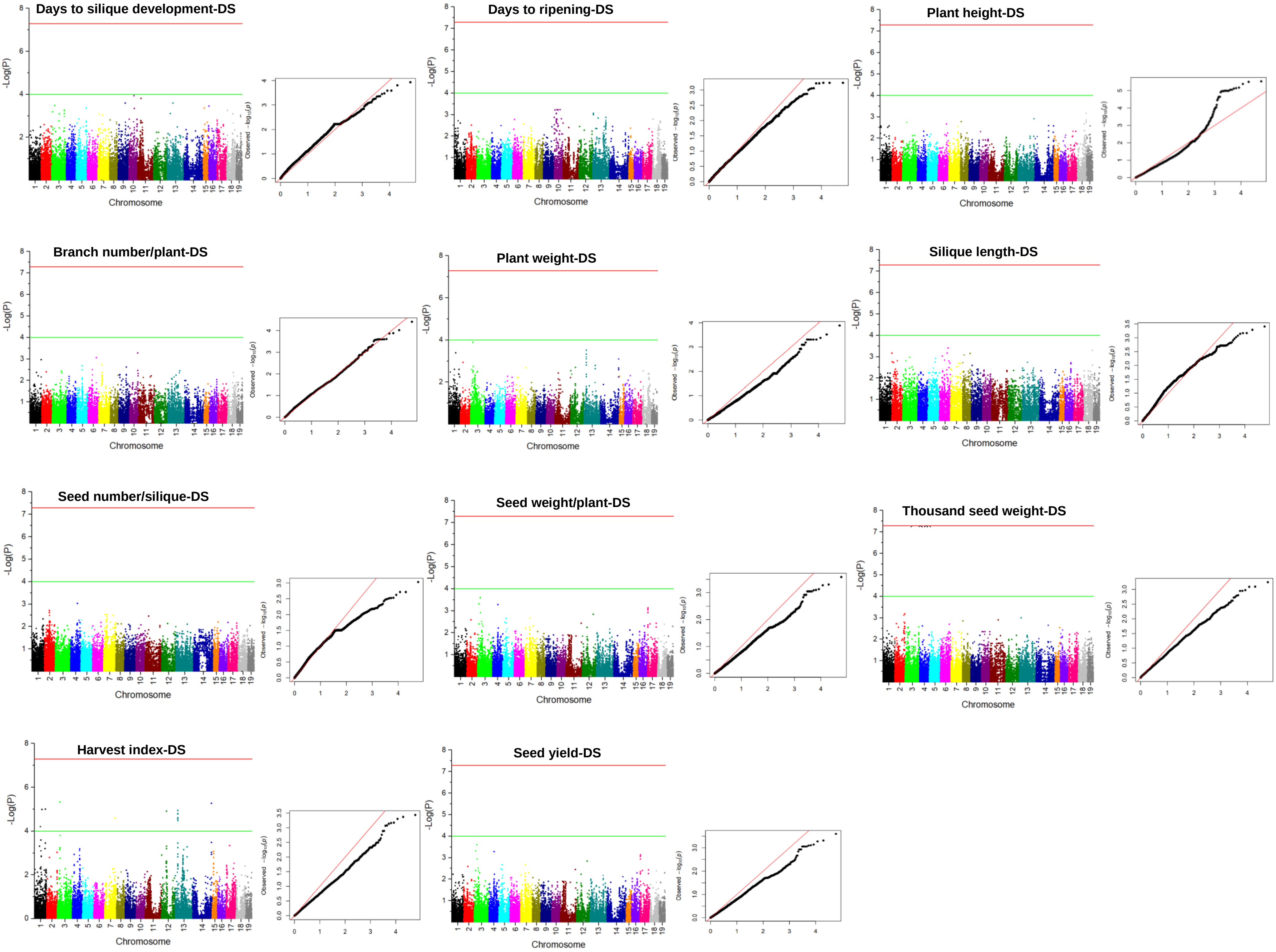
Days to silique development-DS
Days to ripening-DS
Plant height-DS
Branch number/plant-DS
Plant weight-DS
Silique length-DS
Seed number/silique-DS
Seed weight/plant-DS
Thousand seed weight-DS
Harvest index-DS
Seed yield-DS
-Log(P)
Chromosome
Observed −log10(p)

Supplementary Figure S5 Genome-wide association-mapping results for agronomic and yield related traits in rapeseed (*Brassica napus*) using single-trait analysis in all accessions grown in season 2018. Manhattan plot and corresponding quantile-quantile (QQ) plot displaying the GWAS result of agronomic and yield related traits in 19 chromosomes at well-watered (WW) and drought stress (DS) conditions. In the QQ plot, the x-axis displayed the expected –log10 *p*-value and y-axis represented the observed –log10 *p*-value.

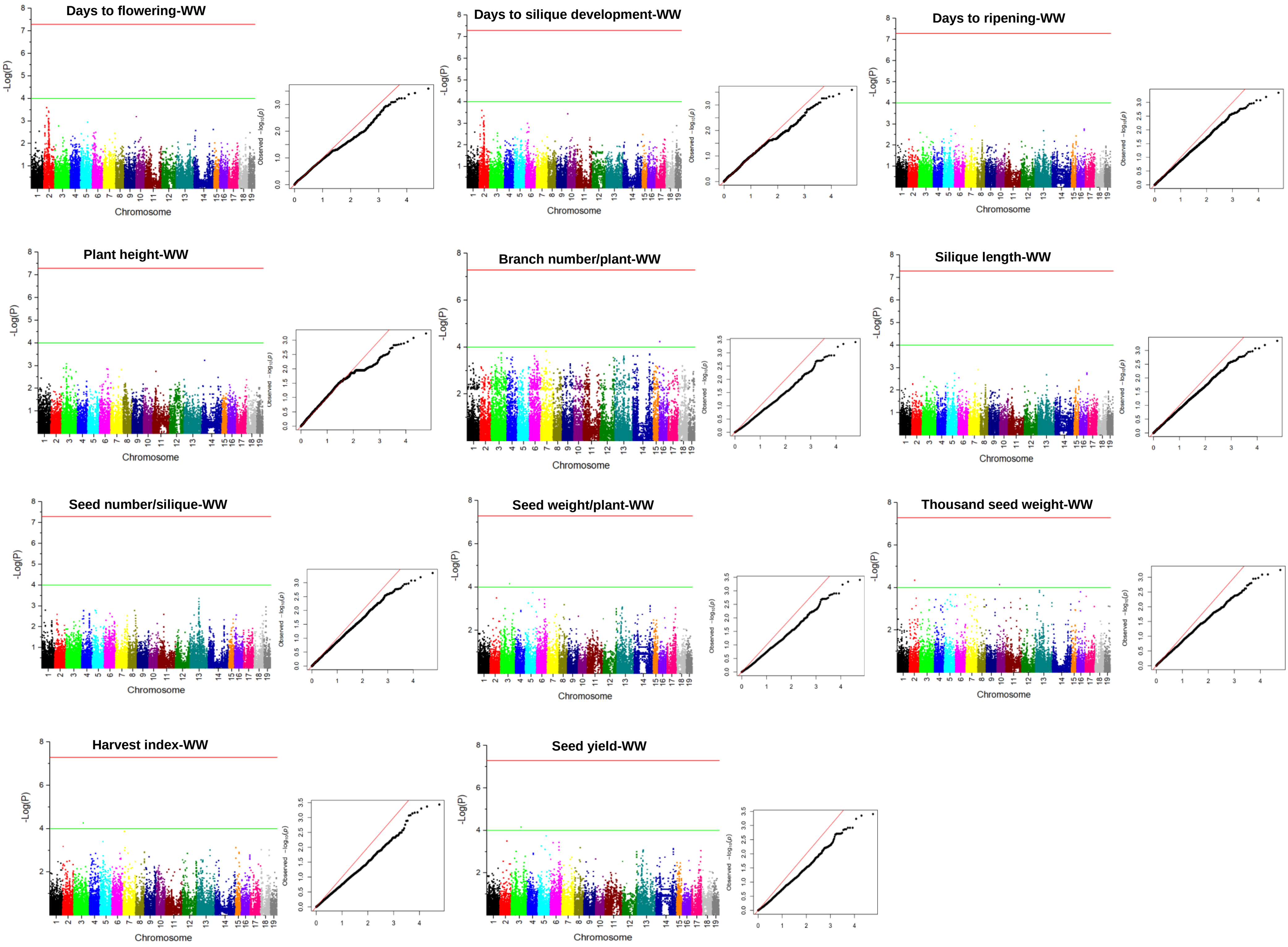
Days to flowering-WW
Days to silique development-WW
Days to ripening-WW
Plant height-WW
Branch number/plant-WW
Silique length-WW
Seed number/silique-WW
Seed weight/plant-WW
Thousand seed weight-WW
Harvest index-WW
Seed yield-WW
-Log(P)
Chromosome
Observed −log10(p)

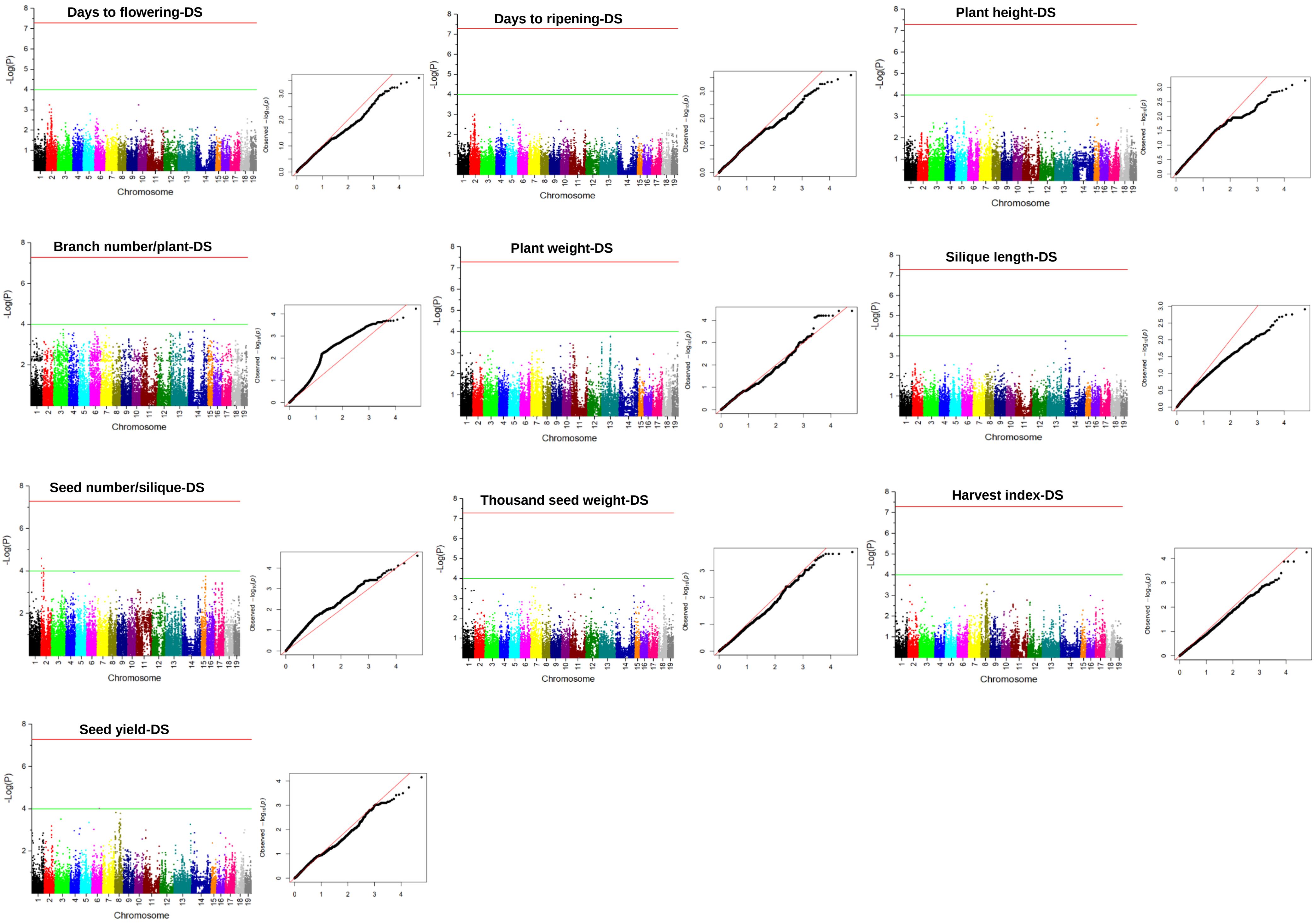
Days to flowering-DS
Days to ripening-DS
Plant height-DS
Branch number/plant-DS
Plant weight-DS
Silique length-DS
Seed number/silique-DS
Thousand seed weight-DS
Harvest index-DS
Seed yield-DS
-Log(P)
Chromosome
Observed −log10(p)

Supplementary Figure S6 Genome-wide association-mapping results for agronomic and yield related traits in rapeseed (*Brassica napus*) using single-trait analysis in all accessions grown in season 2020. Manhattan plot and corresponding quantile-quantile (QQ) plot displaying the GWAS result of agronomic and yield related traits in 19 chromosomes at well-watered (WW) and drought stress (DS) conditions. In the QQ plot, the x-axis displayed the expected –log10 *p*-value and y-axis represented the observed –log10 *p*-value.

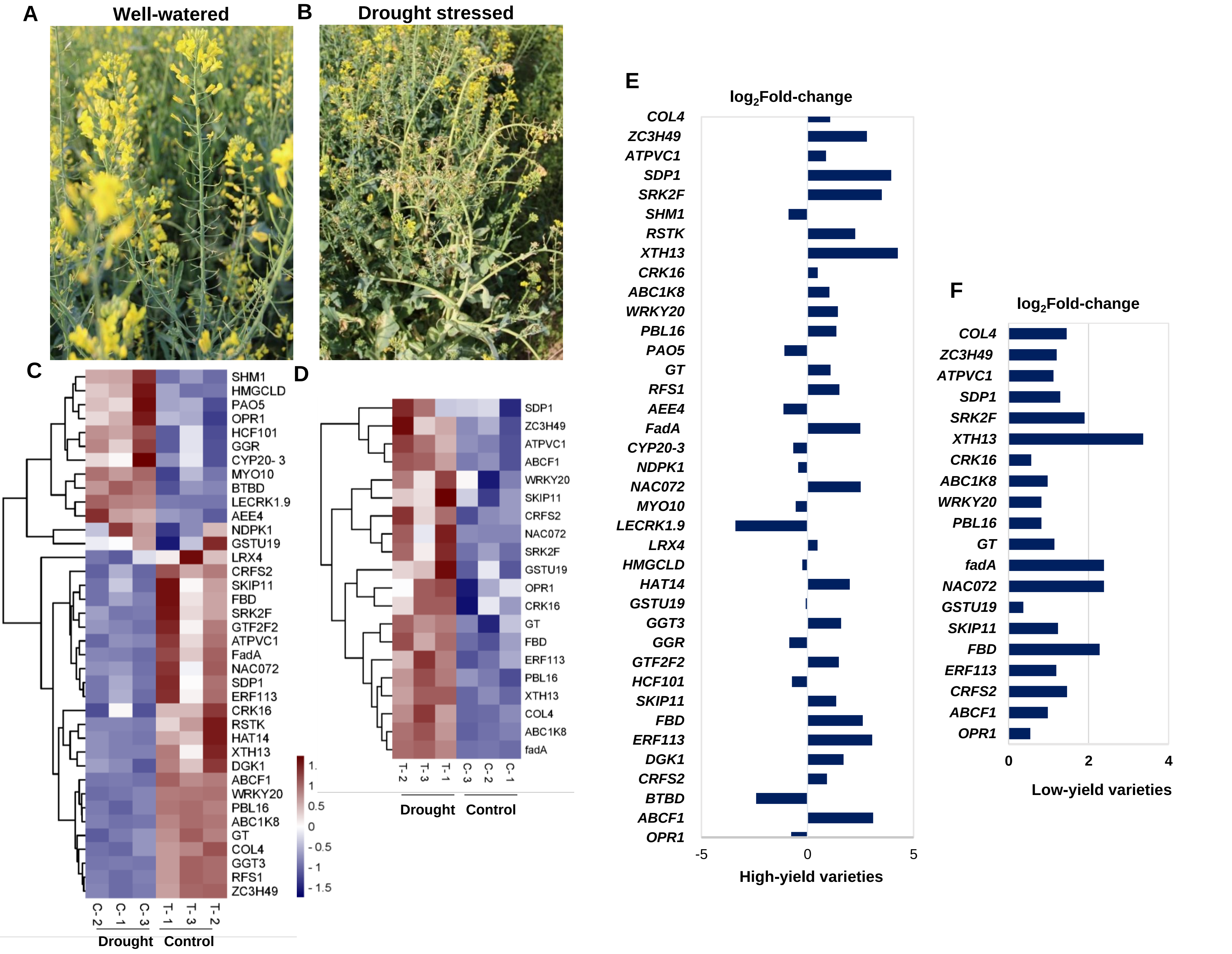
A
Well-watered
B
Drought stressed
C
SHM1
HMGCLD
PAO5
OPR1
HCF101
GGR
CYP20- 3
MYO10
BTBD
LECRK1.9
AEE4
NDPK1
GSTU19
LRX4
CRFS2
SKIP11
FBD
SRK2F
GTF2F2
ATPVC1
FadA
NAC072
SDP1
ERF113
CRK16
RSTK
HAT14
XTH13
DGK1
ABCF1
WRKY20
PBL16
ABC1K8
GT
COL4
GGT3
RFS1
ZC3H49
C-2 C-1 C-3 T-1 T-3 T-2
Drought Control
1.
1
0.5
0
- 0.5
- 1
- 1.5
D
SDP1
ZC3H49
ATPVC1
ABCF1
WRKY20
SKIP11
CRFS2
NAC072
SRK2F
GSTU19
OPR1
CRK16
GT
FBD
ERF113
PBL16
XTH13
COL4
ABC1K8
fadA
T-2 T-3 T-1 C-3 C-2 C-1
Drought Control
E
log2Fold-change
COL4
ZC3H49
ATPVC1
SDP1
SRK2F
SHM1
RSTK
XTH13
CRK16
ABC1K8
WRKY20
PBL16
PAO5
GT
RFS1
AEE4
FadA
CYP20-3
NDPK1
NAC072
MYO10
LECRK1.9
LRX4
HMGCLD
HAT14
GSTU19
GGT3
GGR
GTF2F2
HCF101
SKIP11
FBD
ERF113
DGK1
CRFS2
BTBD
ABCF1
OPR1
-5 0 5
High-yield varieties
F
log2Fold-change
COL4
ZC3H49
ATPVC1
SDP1
SRK2F
XTH13
CRK16
ABC1K8
WRKY20
PBL16
GT
fadA
NAC072
GSTU19
SKIP11
FBD
ERF113
CRFS2
ABCF1
OPR1
0 2 4
Low-yield varieties

Supplementary Figure S7 Transcriptomic analysis of high and low-yield rapeseed varieties under drought stress. (A and B) Responses of the rapeseed plant to drought stress at flowering stage. (A) Rapeseed plant phenotypes under well-watered condition. (B) Rapeseed plant phenotypes at 3 weeks after stress. (C and D) Heatmap of drought-induced genes in high and low-yield varieties under drought stress. (C) Thirty-eight drought-induced genes in high-yield varieties. (D) Twenty drought-induced genes in the low-yield varieties. (E) Bar plot of upregulated and downregulated of key drought-induced genes in high-yield varieties. (F) Bar plot of upregulated and downregulated of key drought-induced genes in low-yield varieties.
